# Genetic Biodiversity of Italian Olives (*Olea europaea*) Germplasm Analyzed by SSR Markers

**DOI:** 10.1155/2014/296590

**Published:** 2014-02-27

**Authors:** Innocenzo Muzzalupo, Giuseppe Giovanni Vendramin, Adriana Chiappetta

**Affiliations:** ^1^Consiglio per la Ricerca e Sperimentazione per l'Agricoltura, Centro di Ricerca per l'Olivicoltura e l'Industria Olearia (CRA-OLI), C.da Li Rocchi-Vermicelli, 87036 Rende, Italy; ^2^Consiglio Nazionale delle Ricerche, Istituto di Bioscienze e BioRisorse, 50019 Sesto Fiorentino, Italy; ^3^Università della Calabria, Dipartimento di Biologia, Ecologia e Scienza della Terra, Ponte P. Bucci, 87036 Arcavacata di Rende, Italy

## Abstract

The olive is an important fruit species cultivated for oil and table olives in Italy and the Mediterranean basin. The conservation of cultivated plants in *ex situ* collections is essential for the optimal management and use of their genetic resources. The largest *ex situ* olive germplasm collection consists of approximately 500 Italian olive varieties and corresponding to 85% of the total Italian olive germplasm is maintained at the *Consiglio per la Ricerca e sperimentazione per l'Agricoltura*, *Centro di Ricerca per l'Olivicoltura e l'Industria Olearia (CRA-OLI)*, in Italy. In this work, eleven preselected nuclear microsatellite markers were used to assess genetic diversity, population structure, and gene flows with the aim of assembling a core collection. The dendrogram obtained utilizing the unweighted pair group method highlights the presence of homonymy and synonymy in olive tree datasets analyzed in this study. 439 different unique genotype profiles were obtained with this combination of 11 loci nSSR, representing 89.8% of the varieties analyzed. The remaining 10.2% comprises different variety pairs in which both accessions are genetically indistinguishable. Clustering analysis performed using BAPS software detected seven groups in Italian olive germplasm and gene flows were determined among identified clusters. We proposed an Italian core collection of 23 olive varieties capturing all detected alleles at microsatellites. The information collected in this study regarding the CRA-OLI *ex situ* collection can be used for breeding programs, for germplasm conservation, and for optimizing a strategy for the management of olive gene pools.

## 1. Introduction

The olive (*Olea europaea* L. subsp. *europaea* var. *europaea*) is an important fruit species cultivated for oil and canned fruit in Italy and the Mediterranean basin. The existing *ex situ* collections of olive tree germplasm may valuably provide either raw material for plant breeding or plants which are directly valid for a sustainable production. With respect to the latter, we refer to those local varieties that evolved for a very long period in a location, that developed adaptative traits which are well integrated with the environmental, agronomic, cultural, and traditional features of the site, and that have been relatively recently replaced with new varieties [1]. The needs of modern agriculture, such as sustainability, call for the cultivation of a wider range of diverse material that could better respond to the different aspects involved. Specifically, if it is necessary to obtain new varieties with a broader genetic base, capable of producing under diverse conditions and of responding to different stresses, that is, drought, pests, low fertility of the soil, and so forth, the reintroduction of old local varieties and the safeguard of traditional farming systems and landscapes can be very profitable from a socioeconomic point of views [2]. In general, the lack of information about plant genetic resources has the effect of limiting their use, restricting both the value and the usefulness of a collection even within the owning institute and among other potential users [3]. Hence, the characterisation of the germplasm conserved in a collection is an essential prerequisite to a proper and wide utilization of the conserved plant material and it is the first step toward the definition of the roles that the varieties can play in sustainable production, through the direct use or in breeding programs [4, 5]. In this respect, several Mediterranean cities have promoted *ex situ* olive germplasm collections, including Cordoba (Spain), Marrakech (Morocco), Porquerolles (France), and Cosenza (Italy), which hosts the majority of olive varieties. Currently, on the basis of estimates from the FAO Olive Germplasm Plant Production and Protection Division, the world olive germplasm contains more than 2.600 different varieties [6], but this number could possibly have been underestimated as there is a significant lack of information regarding minor local varieties and ecotypes that are widespread in different olive-growing areas. An accurate and unambiguous identification of cultivars can be of particular importance, since different olive oils, due to their unique organoleptic and sensorial characteristics, have obtained marks of protected designation of origin (PDO) at a European level according to EC Regulation 2081/92 [7]. The main production of olive oil in Southern Italy is comprised by PDO olive oils, even though many olive cultivars with table purposes are likewise widely grown, since drupe consumption belongs to the Mediterranean diet. Over 750 million olive trees are cultivated worldwide; about 95% of them are to be found in the Mediterranean region. About 80% of the global olive oil production in 2011-2012 came from the European Union, of which 77% is concentrated in Spain, Italy, and Greece (http://ec.europa.eu/agriculture). The European Union, with about 32%, is also the major producer of world's table olives. Even, in this case, the largest producing European countries are Spain, Greece, and Italy. Italy has about 600 olive cultivars and holds the world record for the number of cultivated varieties, representing 25% of the known world olive germplasm [8]. The Italian germplasm is large and variegated on a regional scale, because each region has gradually selected cultivars adapted to local conditions. The largest *ex situ* olive germplasm collection consisting of approximately 500 Italian olive varieties, and corresponding to 85% of the total Italian olive germplasm and to more than 18% of the total world olive germplasm, is maintained at the *Consiglio per la Ricerca e sperimentazione per l'Agricoltura, Centro di Ricerca per l'Olivicoltura e l'Industria Olearia* (CRA-OLI, Agricultural Research Council-Olive Growing and Oil Industry Research Centre) in Italy [6]. The systematic collection of Italian olive varieties for deposit into specific catalogue fields started in Italy during the 1980s. A similar international collection was initiated in 1997 by CRA-OLI of Rende, Italy. Collection included the following steps: a survey of the territory, identification, a basic characterization, and the introduction into the gene bank field. Material identified by other international scientific institutions (International Treaty on Plant Genetic Resources for Food and Agriculture-Plant Genetic Resources RGV-FAO Projects) was also included. To date, about 500 varieties have been introduced into the CRA-OLI collection (http://www.certolio.org/data-base-molecolare/).

However, this wealth in terms of available biodiversity has often generated many complications in olive germplasm classification due to the lack of reference standards and confusion regarding the varietal names, with numerous cases of homonymy (one denomination for several genotypes) and synonymy (one genotype with several denominations) [9, 10]. It therefore appears clear how the characterization of the genetic structure is important in both the management of the olive gene pool and in understanding the role played by the domestication and subsequent crop expansion of olive trees.

The identification of cultivars and accessions using molecular markers is a crucial aim of modern horticulture, with applications in breeding programs and in germplasm collection management. Traditionally olives, like other tree species, were characterized by morphological traits [11]. However, certain limitations associated with these traits have made them less popular in germplasm characterization and diversity analysis. The availability of molecular tools has provided more robust and reliable tools for germplasm characterization.

In recent years, many studies about molecular characterization of germplasm in olive trees have been performed, but generally on a small number of Italian olive cultivars and without taking into account the presence of a core collection [3, 12–15]. A core collection is a subsample of a large germplasm collection that contains the minimum number of individuals that represent the whole genetic diversity and phenotypic variability of the original collection [16], essential to optimise the management and use of the large *ex situ* olive collections.

The purpose of this study was to investigate the genetic structure of the entire Italian olive germplasm CRA-OLI collection, including all 489 accessions, using eleven nuclear microsatellite (SSRs) markers. The genetic structure of Italian olive germplasm was investigated using a model-based Bayesian clustering method to assign individuals into defined gene pools. This work is the first that takes into account such a large number of Italian olive cultivars (489 cvs) analyzed using the same set of molecular markers. This study also provides basic information for the development of core collections to maximise the representativeness of olive genetic diversity. Our results represent an essential step towards optimised conservation of olive genetic resources and subsequently for genetic association studies to detect quantitative trait loci (QTL) of adaptive and agronomic interest [17].

## 2. Materials and Methods

### 2.1. Olive Germplasm Collection

Young leaves were harvested from 489 olive trees growing in the germplasm collection of CRA-OLI, located along the Ionian coasts near Mirto-Crosia (Calabrian region, Southern Italy). The analyzed olive plants are all autochthonous and representative of the seventeen regions of Italy: Abruzzo (23 varieties), Basilicata (29), Calabria (36), Campania (43), Emilia-Romagna (12), Friuli-Venezia-Giulia (3), Lazio (25), Liguria (16), Lombardy (2), Marche (19), Molise (24), Apulia (41), Sardinia (20), Sicily (70), Tuscan (101), Umbria (22), and Veneto (3) (see Table S1 in Supplementary Material available online at http://dx.doi.org/10.1155/2014/296590).

### 2.2. DNA Extraction and Microsatellites Analysis

Total genomic DNA was isolated from 100 mg fresh leaves, previously ground in liquid nitrogen, using the PureLink Genomic Plant DNA Purification Kit (Invitrogen, California, USA). DNA quality was checked on 0.9% agarose gel and the DNA concentration was estimated using NanoDrop ND2000 spectrophotometer (Thermo Scientific, Massachusetts, USA).

The olive trees were genotyped at 11 nuclear SSRs, selected among those available in literature, and proven to be suitable for the characterization and identification of olive varieties in previous papers [10, 14, 18]: four SSRs (GAPU59, GAPU71A, GAPU71B, and GAPU103A) by Carriero et al. [19], five (UDO01, UDO03 UDO12, UDO28, and UDO39) by Cipriani et al. [20], and two (DCA9 and DCA18) by Sefc et al. [21].

The PCR was conducted in a final volume of 25 *μ*L containing 25 ng of DNA, 10 mM Tris-HCl pH 8.0, 1.5 mM MgCl_2_, 0.2 mM dNTPs, 0.25 *μ*M forward and reverse primers, and 0.05 units of Taq DNA polymerase (Invitrogen, California, USA) as reported in Muzzalupo et al. [22]. SSR amplification was carried out as described by Muzzalupo et al. [14]. The amplification products were analyzed by means of a 2100 Bio-Analyzer instrument (Agilent Technologies, Waldbronn, Germany) with the 2100 BioSizing software (version A.02.12) using DNA 500 LabChip kit. To assign the correct size to alleles, most alleles of the selected loci were sequenced. The allele sequencing was carried out as described by Baldoni et al. [18].

### 2.3. Data Analysis

#### 2.3.1. Genetic Diversity and Multivariate Analysis

Number of alleles (*Na*), effective number of alleles (*Ne*), observed (*Ho*) and expected (*He*) heterozygosity, and fixation index (*F*) were computed with GenALEx version 6.5 software [23].

The alleles detected for each microsatellite were recorded into a data matrix of presence (1) and absence (0) of bands (each allele representing a band). Genetical distance based on the Nei coefficient and genetic similarity based on the Simple Matching (SM) coefficient among 489 olive varieties were estimated using the NTSYSpc program version 2.02 [24]. Finally, a tree was inferred using the unweighted pair group method using an Arithmetic average (UPGMA) clustering algorithm to highlight the presence or absence of synonymies in the olive varieties data set analyzed in this study. In addition, the dendrogram was tested by bootstrapping to determine the confidence limits and using WinBoot program [25].

The frequency of null alleles was estimated per locus and per region, using the software FreeNA [26].

Principal Coordinate Analysis (PCoA), also available in version 6.5 of the GenALEx program, was conducted using Nei's unbiased genetic distance pairwise population matrix to determine whether observed patterns in molecular data support the partitioning of the olive tree samples into specific groupings.

#### 2.3.2. Bayesian Model-Based Clustering Analysis, Molecular Variance, and Gene Flows

To study the genetic structure of the Italian olive germplasm, a model-based analysis was performed using BAPS 5.3 [27]. This program uses both a nonspatial and spatial Bayesian clustering algorithm assignment to determine the number of genetically distinct populations present in a sample based on allele frequencies. We conducted admixture and mixture analysis on olive varieties distributed at the regional level and using the nonspatial model. BAPS was run setting 1000 as the number of interactions used to estimate the admixture coefficients for the genotypes, 200 as the number of reference individuals from each genotype, and 10 as the number of interactions used to estimate the admixture coefficients for the reference individuals, reanalyzing and comparing our data set also using smaller (5) and higher (20) values.

In addition, an Analysis of Molecular Variance (AMOVA) [28, 29] was performed to estimate levels of genetic differentiation by computing Φ_PT_, *F*_ST_, and *R*_ST_ estimators among BAPS groups identified in this study. Statistical significance of all the Φ_PT_, *F*_ST_, and *R*_ST_ estimators were tested using 10,000 permutations.

Finally, we used the GraphViz 2.28 package installed in BAPS 5.3, to estimate and draw the gene flows among the clusters identified. In the graph, gene flows were shown by weighted arrows, so that the weights relating to the amounts of ancestry in the source cluster were assigned to the target cluster. This step was performed using the result file from the dataset admixture analysis and by setting the threshold for the significance of *P* values of the admixture estimates to 0.05.

#### 2.3.3. Core Collection Sampling

The Maximisation strategy [30–32] implemented in the COREFINDER software [33] was used to generate core olive collection that maximised the number of observed alleles in our nuclear dataset. The M-strategy consists in detecting the best sample size that captures 100% of the genetic diversity present within the entire germplasm collection. The algorithm is based on the Set-Covering (NP-complete) problem. The procedure is a Las Vegas style randomized algorithm: an iteration number is provided by the user, and the algorithm, starting from a random initial set, uses a greedy strategy to search for an accession “A” providing a better overall genetic diversity than some accession “B” belonging to the current core collection. In such a hypothesis, “A” is included and “B” is excluded from the collection. The greedy step is performed exhaustively and each iteration starts with a different initial random set, thereby reducing the probability of ending in a local maximum. In our COREFINDER analysis, the algorithm parameters were set on 100 and 1.000.000 for interations and random seed, respectively.

## 3. Results

### 3.1. Genetic Diversity

Eleven published primer pairs flanking nuclear microsatellites were employed to investigate the level of genetic variation among the 489 Italian olive varieties analyzed in this study and present in the olive germplasm collection of the CRA-OLI. A total of 84 alleles over 11 loci were detected, ranging from 3 at UDO01 locus to 12 alleles at both UDO39 and DCA9 loci. The shortest allele among the 11 polymorphic loci was allele 108 base pairs (bp) at UDO39, whereas the longest was 259 bp at GAPU71A (Table S2). The most common size variant, namely, the allele 214 bp at locus GAPU71A, was found with a frequency of 0.485 and showed the highest value in the varieties within Molise region (0.813). Three out of 84 alleles were considered private alleles, since they were present only in “Cellina di Nardò” (allele 228 bp at locus GAPU71A), in “Arancino,” “Ciliegino,” “Emilia,” “Grappolo,” “Gremignolo,” “Gremignolo di Bolgheri,” “Lastrino,” “Lazzera reale,” “Salicino,” “Santa Caterina,” “Borgiona,” and “Corniolo” (allele 156 bp at locus UDO12), and in “Filare” (allele 184 bp at locus DCA9) with a frequency <1%.

The effective number of alleles (*Ne*) ranged from 2.3 to 6.7 with a mean of 4.3 (Table 1).

In all the studied varieties, the observed heterozygosity (mean *Ho* = 0.605) was lower than expected (mean *He* = 0.664). The difference determines a significant positive value for the mean fixation index (*F* = 0.114) that could be attributed to the presence of null alleles (Table 1).

Therefore, FreeNA software [26] was used to estimate the null allele frequencies. Values >0.20 of null allele frequency have been considered as a threshold over which a significant underestimation of *He* due to null alleles can be found. Frequencies lower than 0.20 were obtained for all loci for each of the sampled varieties, except for UDO01 (0.297), UDO03 (0.295), and UDO39 (0.214) loci, where null allele frequencies higher than 0.20 were found (Table 1). For this reason, these loci were eliminated from further analysis.

### 3.2. Characterisation of Olive Accessions

The dendrogram (Figure S1) obtained utilizing the UPGMA method that elaborates a matrix of similarity obtained using NTSYSpc program version 2.02 [24] was tested using WinBoot program [25] and highlights the presence or absence of mislabeling, redundancies, homonymy, and synonymy in olive tree datasets analyzed in this study. 439 different unique genotype profiles were obtained with this combination of 11 loci, being able to identify about 89.8% of the varieties analyzed showing unique profiles. The remaining 10.2% is comprised of different variety pairs in which both accessions are genetically indistinguishable one from another, which has the potential to represent cases of synonymy (Table 2). Synonyms included cultivars with the same profile for all SSR examined and cultivar pairs differing from each other for one or two alleles [14, 34, 35]. Ten different olive cultivar pairs or groups are genetically indistinguishable from one another (Table 2). Many of these possible cases of synonymy are in agreement with previous studies based on morphological descriptors and molecular marker systems [10, 14, 15, 22, 36]; others were encountered for the first time.

Parent-offspring relations were found for “Giarraffa” and “Pizzo di Corvo,” “Nera di Oliena” and “Paschixedda,” “Cazzarella” and “Sperone di Gallo,” “Grossa di Venafro” and “Paesana Nera,” “Paesana Bianca” and “Rosciola di Rotello,” “Racemo 1” and “Coratina,” “Rossina” and “Selvatico,” and “Toccolana” and “Olivetta nera,” and these eight cultivar pairs differ by only one allele (Table 2). Parent-offspring relations were found for “Ascolana dura” and “Ascolana semitenera,” “Dolce di Andria” and “Termite di Bitetto,” “Paschixedda” and “Terza Piccola,” “Gentile nera di Colletorto” and “Noccioluta,” “Ginestrino” and “Maurino 2,” “Ginestrino” and “Maurino 4,” “Maurino 2” and “Maurino 4,” “Leccio del Corno 2” and “Piangente 3,” “Nerba” and “Olivo di Castiglione,” and “Nostrale di Fiano Romano” and “Raza.” These ten cultivar pairs differ by two alleles.

Furthermore, 22 cases of homonymy were identified. These 22 cases of homonymy can be divided into two groups according to the number of different alleles. The first group is represented by plants that have a number of different alleles less than ten. The list of homologies is shown in Table 2. A special case is that presented in the group of “Leccino,” “Moraiolo,” “Pendolino,” “Maurino,” “Nostrana di Brisighella,” and “San Felice Acquasparta” which, in previous work, had been regarded as polyclonal varieties [22, 37]. In fact, the “Leccino” group is represented by 10 plants that clustered forming subgroups with microsatellite profiles that differ by a minimum of one allele to a maximum of eight alleles. A similar pattern was observed in the case of the group of “Moraiolo” (4 plants), of “Maurino” (4), of “Pendolino” (5), and “Nostrale di Brisighella” (4) that show differences in the profile microsatellite, from 1 to 3 alleles, from 1 to 2 alleles, and from 3 to 9 alleles, respectively. Finally, the “San Felice Acquasparta” denomination is represented by five plants that do not cluster. Only plants 4 and 5 formed subgroups with the same SSR profiles; however, they were differentiated from plants 1, 2, and 3 by a minimum of nine to a maximum of ten alleles.

Finally, SSR analysis allowed the classification of the CRA-OLI olive germplasm into 439 unique molecular profiles corresponding to well-defined genotypes, whereas, for the remaining molecular profiles, they reveal the presence of accessions considered as clones or possible synonyms of the same genotype.

### 3.3. Genetic Structure of Italian Olive Genotypes

**Figure 1:**
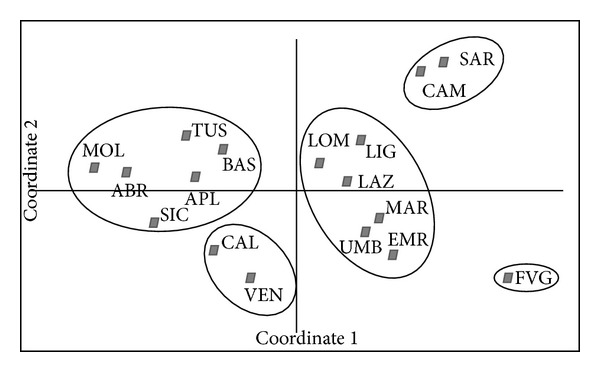
Principal Coordinates Analysis (PCoA) plot of the olive varieties based on the first two principal coordinates (coord. 1 = 54.43% and coord. 2 = 14.52%). Legend: ABR: Abruzzo, APL: Apulia, BAS: Basilicata, CAL: Calabria, CAM: Campania, EMR: Emilia-Romagna, FVG: Friuli-Venezia-Giulia, LAZ: Lazio, LIG: Liguria, LOM: Lombardy, MAR: Marche, MOL: Molise, SAR: Sardinia, SIC: Sicily, TUS: Tuscany, UMB: Umbria, and VEN: Veneto.

Genetic structure was tested using two different approaches. First, the PCoA, performed on Nei's unbiased genetic distance matrix and, based on 62 different size variants, showed that the 439 olive varieties were separated into five main groups (Figure 1).

Group I contains olive varieties of Molise (23), Tuscany (79), Abruzzo (23), Basilicata (28), Apulia (35), and Sicily (64). Group II comprises Calabria (34) and Veneto (3) varieties. Group III include the olive varieties present in the regions of Lombardy (1), Lazio (24), Liguria (12), Marche (19), Umbria (21), and Emilia-Romagna (12), while Group IV contains varieties of Campania (42) and Sardinia (16). Group V only contains varieties from Friuli-Venezia-Giulia (3) region and is slightly more distant from genetic groups previously described. In the PCoA analysis, the first two principal axes explain a total of 68.9% of unbiased genetic distance, with 54.4% and 14.5% for coordinates 1 and 2, respectively (Figure 1).

**Figure 2:**
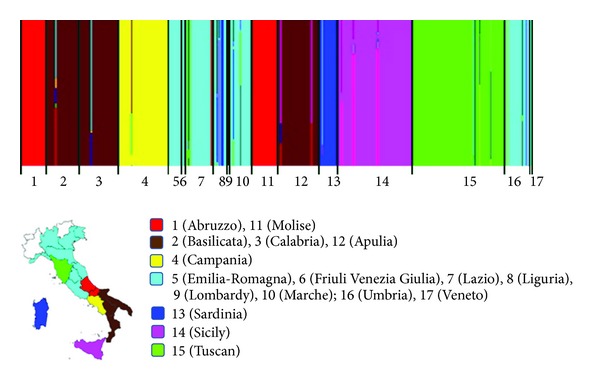
BAPS analysis on the 439 Italian olive varieties. In the graph, each colour represents a population group based on allele frequency. Vertical bars represent each olive variety analyzed in this study and bars are divided into several colours when there is evidence of admixture.

Bayesian clustering algorithms, such as those implemented in BAPS 5.3 program, were used for inferring olive Italian germplasm structure [27]. Seven genetic clusters were identified with a more specific distribution of genotypes than PCoA analysis. The identified clusters are represented in Figure 2.

Our analysis showed that all the olive varieties present in Northern and Central Italy were grouped in a single genetic cluster, with the exception of the varieties present in the regions of Tuscany, Abruzzo, and Molise that were grouped into two separate clusters (Figure 2). The BAPS analysis also revealed how the varieties distributed in the remaining regions of Southern Italy (Campania, Apulia, Calabria, and Basilicata), as well as those present in the two major islands of Sicily and Sardinia, are grouped into four distinct genetic clusters (Figure 2).

Furthermore, BAPS analysis showed high levels of *Ho* in each genetic cluster identified and ranged from 0.680 to 0.852 with a mean of 0.755, than *He* levels (mean 0.724) producing a negative value of *F* (mean −0.045) (Table 3).

In addition, the AMOVA analysis revealed comparable values of *F*_ST_ and *R*_ST_ estimators among BAPS groups (*F*_ST_ = 5.7 and *R*_ST_ = 5.2), with a higher Φ_PT_ estimator value (Φ_PT_ = 10.9) than those reported in literature for *Olea* [37]. All of the AMOVA analysis conducted as part of this study showed that most of the diversity being expressed within BAPS groups identified for all the estimators is considered (Table 4).

### 3.4. Gene Flows

**Figure 3:**
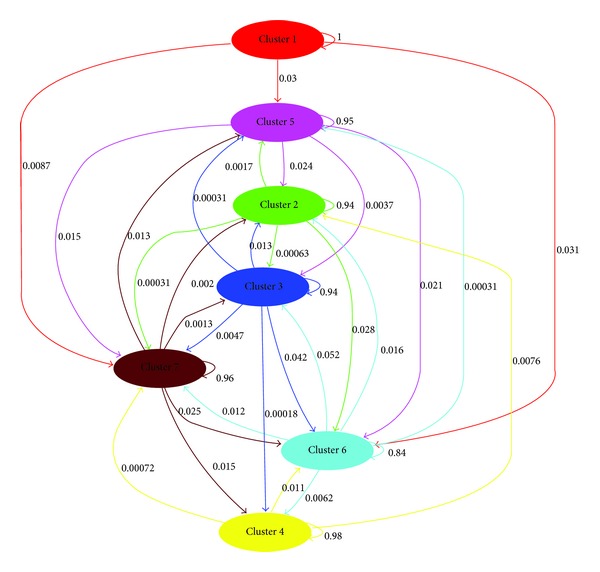
Main gene flows between clusters identified in Italian olive germplasm collection of CRA-OLI. In the graph, gene flows were shown by weighted arrows, so that the weights relative amounts of ancestry in the source cluster were assigned to the target cluster.

The analysis of the CRA-OLI Italian olive germplasm collection performed with a Bayesian clustering software, demonstrated a good network of gene flows between the clusters identified in this study. This analysis also revealed how only the clusters that grouped the olive varieties present in Sicily (64), Tuscany (79), Sardinia (16), Apulia (35), Basilicata (28), and Calabria (34) and those included in Northern and Central Italy were more susceptible to the identified gene flows, with a consequent transfer of genetic material (Figure 3). The analysis of gene flows showed that clusters 3 (Sardinia), 6 (Emilia-Romagna, Friuli-Venezia-Giulia, Lazio, Liguria, Lombardy, Marche, Umbria, and Veneto), and 7 (Basilicata, Calabria, and Apulia) were characterized by a higher level of output gene flow, while only clusters 6 and 7 had a very high level of input genetic material transfer. On the other hand, this analysis showed that, only in cluster 1 (Abruzzo and Molise), no input gene flows with other clusters seemed to occur.

### 3.5. Core Collection

**Figure 4:**
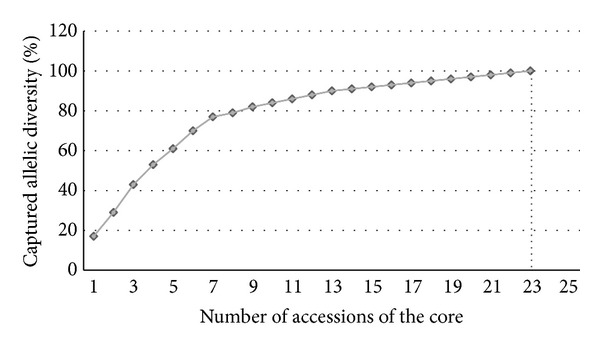
Genetic diversity as a function of the number of accessions included in the Italian olive germplasm core collection.

A core collection was herein assembled for Italian olive germplasm, aiming to represent the entire genetic diversity identified in this study. The COREFINDER analysis based on M-strategy showed that, for Italian olive germplasm, 100% of the SSR alleles found in this study could be represented by a core collection of 23 accessions (Figure 4 and Table S3). In addition, our COREFINDER analysis highlighted that 39% of the entire core collection was represented by the olive varieties grouped in cluster 1 (Abruzzo and Molise) identified by BAPS analysis. Other BAPS clusters contribute to the core collection at smaller percentages: cluster 6 (26%), cluster 2 (13%), clusters 4 and 7 (9%), and cluster 3 (3%). Overall, the core collection identified in this study represents 5.2% of the CRA-OLI olive germplasm collection.

## 4. Discussion

The results by SSR analysis of CRA-OLI Italian olive germplasm collection show abundant allelic variation over 11 loci and high overall genetic diversity, confirming that SSR markers can be effectively used to genotype a germplasm collection. Four of these loci were included in the best consensus set of SSR markers [18] that has already been used for genetic structure studies [14].

It is currently well known how mating systems play a key role in determining the structure of genetic diversity in natural and domesticated genotypes. This is especially true for olive trees that have been clonally propagated since ancient times. This claim was also confirmed in our study by clustering analysis, NTSYS, and PCoA, performed on our dataset. For NTSYS analysis, the results demonstrated the presence of synonyms and homonyms among the different varieties in the Italian olive germplasm which are partially comparable to those reported in literature. Homonymy and synonymy characterization is essential in order to avoid genotype redundancy and to maximize genetic diversity in the Italian olive germplasm collection. Additionally, the PCoA analysis showed a clear grouping of the olive Italian varieties into five main clusters, broadly confirming previously reported results [12–14].

Bayesian analysis (BAPS) further provided support for the existence of genetic structure in CRA-OLI germplasm collection and separated the Italian olive varieties into seven main clusters. The Bayesian model-based analysis highlights the real structure and distribution of Italian olive germplasm gene pools, separating the major genetic cluster 6 that grouped olive varieties in Northern and Central Italy, from the other six gene pools found. In addition, BAPS analysis results show the genetic relationship, represented by gene flows, among the seven clusters identified, confirming that the current gene pools and distribution of Italian olive germplasm are due to geographic and cultural aspects mainly involving human activity in the past.

Moreover, the AMOVA results surprisingly revealed a higher Φ_PT_ estimator value (Φ_PT_ = 10.9) than those reported in literature for *Olea europaea* [37], showing a good level of genetic differentiation distributed at a genetic cluster level among Italian olive varieties in the CRA-OLI collection.

This result was confirmed by higher levels of *Ho* and *He* detected in BAPS clusters, with a mean negative value of *F* that clearly highlights the good levels of genetic diversity, maintained costant in each BAPS group identified in this study.

The final aim of the study was to construct a valid core collection for cultivated olives in the CRA-OLI germplasm collection, sampling the minimum number of entries that maximize the representativeness of allelic diversity. The core collection that we proposed in this study consists of 23 varieties that capture 100% of the base collection. It was found that only a small number of olive varieties, compared with other values reported in literature for *Olea* [3], are necessary to represent the molecular diversity revealed in this study. These results are probably due to the percentage of cluster 1 representativeness (39%) within the core collection, further confirming the genetic peculiarity of this cluster, already highlighted by gene flow analysis. Nevertheless, high levels of heterozygosity observed in Italian olive germplasm may contribute to reducing the size of the core collection [33]. Van Hintum [38] suggested that the sampling proportion should vary between 5 and 20% of the base collection, representing at least 70% of overall genetic diversity. The core collections for Italian olive germplasm proposed here represent 100% of the molecular diversity found in this study, with the number of varieties accounting for 5.2% of the CRA-OLI germplasm collection.

Finally, the result of our BAPS analysis supports both NTSYS and PCoA results, demonstrating how all olive accessions analyzed in this study and maintained in CRA-OLI *ex situ* collection could be considered representative of Italian olive germplasm because each genetic group defined in BAPS reflects geographic distribution and confirms that the Italian olive germplasm is a peculiar gene pool present in the Mediterranean basin.

Concluding, the use of molecular markers, like microsatellite, is imperative to build a database for cultivar analysis, for the traceability of processed food, and for the appropriate management of olive germplasm collections. Moreover, the results presented here regarding clustering and core collection are extremely useful for the selections of parents to be used for breeding programs and thus ensuring an optimal management of the CRA-OLI Italian olive germplasm collection. This work is the first study with such a large number of Italian olive varieties analyzed using the same set of molecular markers which allowed characterisation of the genetic structure and identification of a core collection in the largest Italian *ex situ* germplasm collection.

## Supplementary Material

The electronic supplementary material of the research article “Genetic biodiversity of Italian olives (*Olea europaea*) germplasm analyzed by SSR markers” by Muzzalupo et al. is composed of three tables.
Table S1: List of the *Olea europaea* L. varieties present in the Agricultural Research Council-Olive Growing and Oil Industry Research Centre (CRA-OLI) germplasm collection and analyzed by eleven microsatellite markers. The Italian olive varieties included in the core collection are indicated in bold.
Table S2: Alleles detected at the eleven SSR loci in the 489 olive trees analyzed. For each allele, the size and the frequency are reported.
Table S3: Genotype profiles obtained from the combination of the eleven microsatellite markers on 489 olive varieties. The Italian olive varieties included in the core collection are indicated in bold.

## Figures and Tables

**Table 1:** Genetic diversity parameters estimated for the SSR loci in the 489 olive varieties. For each locus, the number of alleles detected (*Na*), the effective number of alleles (*Ne*), the observed (*Ho*) and expected (*He*) heterozygosity, the fixation index (*F*), and the frequency of null allele (*Nu*) are reported.

Locus	*Na*	*Ne*	*Ho*	*He*	*F*	*Nu*
GAPU59	5	4.0	0.632	0.637	0.013	0.042
GAPU71A	9	3.1	0.578	0.602	0.018	0.051
GAPU71B	5	3.6	0.885	0.684	−0.300	0.002
GAPU103A	8	5.7	0.786	0.755	−0.068	0.034
UDO01	3	2.3	0.074	0.504	0.860	**0.297**
UDO03	6	3.2	0.083	0.543	0.857	**0.295**
UDO12	6	3.9	0.828	0.671	−0.260	0.001
UDO28	9	5.6	0.793	0.735	−0.106	0.036
UDO39	12	5.3	0.330	0.704	0.544	**0.214**
DCA09	12	6.7	0.903	0.785	−0.153	0.002
DCA18	9	4.0	0.760	0.686	−0.116	0.008

Mean	7.6	4.3	0.605	0.664	0.114	—
SE	—	0.090	0.024	0.010	0.035	—

The presence of null alleles are indicated in bold.

**Table 2:** Potential cases of mislabelling, redundancies, homonymy, and synonymy identified by microsatellite fingerprinting on 489 Italian olive varieties.

Possible cases of synonyms	Genotype
“Carolea Cefaly”—“Carolea Mirto”	*1 *
“Carolea Cetraro”—“Carolea Rossi”	*2 *
“Cima di Mola”—“Ogliarola salentina”	*3 *
“Leccino Dwarf”—“Leccino Minerva” (plant 1)—“Leccino Pisa” (plants 4 and 9)	*4 *
“Majorca”—“Manna”	*5 *
“Mele”—“Nolca”	*6 *
“Nera di Gonnos”—“Tonda di Cagliari”	*7 *
“Nera di Oliena”—“Terza piccola”	*8 *
“Nera di Villacidro”—“Terza grande”	*9 *
“Ogliarola del Bradano” (plant 1)—“Taggiasca”—“Casaliva”—“Ogliarola barese”—“Ogliarola garganica”—“Correggiolo”—“Correggiolo Montegridolfo”—“Correggiolo Pallese”—“Frantoio” (plants 1/7 − 10)—“Frantoio FC”—“Frantoio Villa Verrucchio”—“Frantoio Montegridolfo”	*10 *

Plants with the same genotypes (G)	

“Arnasca” (plants 1, 2, 3, and 4)	“Arnasca G”
“Buscionetto” (plants 2 and 3)	“Buscionetto G”
“Canino” (plants 1 and 2)	“Canino G”
“Coratina” (plants 1, 2 and 3)	“Coratina G”
“Frantoio” (plants 8 and 9) “Gentile di Larino” (plants 1 and 5)	“Frantoio G” “Gentile di Larino G”
“Iacona” (plants 1 and 2)	“Iacona G”
“Leccino” (plants 3 and 4)	“Leccino G1”
“Leccino” (plants 6, 7, 8, and 9)	“Leccino G2”
“Maiatica di Ferrantina” (plants 1 and 2)	“Maiatica Ferrantina G”
“Moresca” (plants 1 and 2)	“Moresca G”
“Nocellara del Belice” (plants 1 and 2)	“Nocellara del Belice G”
“Nocellara Nissena” (plants 1 and 2)	“Nocellara Nissena G”
“Pirunara” (plants 1 and 2)	“Pirunara G”
“Rotondella di sanza” (plants 1 and 2)	“Rotondella di sanza G”
“San Felice Acquasparta” (plants 4 and 5)	“San Felice Acquasparta G”

Possible cases of synonyms: plants with different genotypes (one allele)	

“Cazzarella” and “Sperone di gallo”	
“Giarraffa” and “Pizzo di corvo”	
“Grossa di Venafro” and “Paesana nera”	
“Leccino 1” and “Leccino 10”	
“Maurino” (plants 1 and 3)	
“Moraiolo” (plants 1 and 3)	
“Moraiolo” (plants 2 and 4)	
“Nera di Oliena” and “Paschixedda”	
“Nostrana di Brisighella” (plants 2 and 4)	
“Paesana bianca” and “Rosciola di Rotello”	
“Pendolino” (plants 2 and 5)	
“Racemo 1” and “Coratina” (plants 1, 2, and 3)	
“Rossina” and “Selvatico”	
“Toccolana” and “Olivetta nera”	

Possible cases of synonyms: plants with different genotypes (two alleles)	

“Ascolana dura” and “Ascolana semitenera”	
“Dolce di Andria” and “Termite di Bitetto”	
“Gentile nera di Colletorto” and “Noccioluta”	
“Ginestrino” and “Maurino” (plants 2 or 4)	
“Leccio del Corno 2” and “Piangente 3”	
“Maurino” (plants 2 and 4)	
“Moraiolo” (plants 4 and 5)	
“Nerba” and “Olivo di Castiglione”	
“Nostrale di Fiano Romano” and “Raza”	
“Paschixedda” and “Terza piccola”	
“Pendolino” (plants 1 and 5)	
“Pendolino” (plants 3 and 4)	

Homonyms: plants with different genotypes (from three to nine alleles)	

“Buscionetto 1” and “Buscionetto G”	
“Cucca” (plants 1 and 2)	
“Erbano” (plants 1 and 2)	
“Faresana” (plants 1 and 2)	
“Leccino G1” and “Leccino G2”	
“Leccino 2” and “Leccino G1”	
“Leccino” (plants 2 and 10)	
“Leccino Minerva” (plants 1 and 2)	
“Moraiolo” (plants 1 and 5)	
“Nostrana di Brisighella” (plants 1 and 3)	
“Ogliarola messinese” (plants 1 and 2)	
“Pendolino” (plants 1 and 3)	
“Piangente” (plants 1 and 3)	
“Pizzo di corvo” (plants 1 and 2)	
“Razzo” (plants 1 and 2)	
“Turdunazza antimosca” (plants 1 and 2)	
“Vallanella” (plants 1 and 2)	

Homonyms: plants with different genotypes (ten or more alleles)	

“Arnasca 4” and “Arnasca G”	
“Giarraffa” (plants 1 and 2)	
“Leccio del Corno” (plants 1 and 2)	
“Minuta” (plants 1 and 2)	
“Minuta” (plants1 and 3)	
“Minuta” (plants 2 and 3)	
“Ogliarola del Bradano” (plants 1 and 2)	
“Piangente” (plants 1 and 2)	
“Racioppa” (plants 1 and 2)	
“Romanella” (plants 1 and 2)	
“San Felice Acquasparta” (plants 1 and 2)	
“San Felice Acquasparta” (plants 2 and 3)	
“San Felice Acquasparta 3” and “San Felice Acquasparta G”	
“Sargano” (plants 1 and 2)	

**Table 3:** Genetic diversity parameters at SSR loci estimated in BAPS groups identified in this study. For each cluster, the observed heterozygosity (*Ho*), the expected heterozygosity (*He*), and the fixation index (*F*) are reported.

BAPS clusters	*Ho*	*He*	*F*
Cluster 1	0.734	0.700	−0.062
Cluster 2	0.725	0.737	0.018
Cluster 3	0.852	0.710	−0.208
Cluster 4	0.762	0.750	−0.017
Cluster 5	0.680	0.665	−0.017
Cluster 6	0.796	0.763	−0.046
Cluster 7	0.736	0.744	0.015

Mean	0.755	0.724	−0.045
SE	0.020	0.012	0.069

**Table 4:** AMOVA analysis for the partitioning of SSR variation of olive varieties among and within BAPS groups identified in this study.

Estimators	Source of variation	df	Variance components	Percentage total variance	*P* value
Φ_PT_Φ_PT_	Among groups	6	0.709	10.9	*P* < 0.001
	Within groups	432	5.814	89.1	

*F*_ST_	Among groups	6	0.177	5.7	*P* < 0.001
	Within groups	871	2.944	94.3	

*R*_ST_	Among groups	6	68.279	5.2	*P* < 0.001
	Within groups	871	1234.244	94.8	

## Abbreviations

AMOVA: Analysis of Molecular Variance
BAPS: Bayesian Analysis of Population Structure
*F*: Fixation index
*F*_ST_: *F*-statistics
GenAlEx: Genetic Analysis in Excel
*He*: Expected heterozygosity
*Ho*: Observed heterozygosity
*Na*: Observed number of alleles
*Ne*: Effective number of alleles
NTSYSpc: Numerical Taxonomy and Multivariate Analysis System
*Nu*: Null allele
nSSR: Nuclear Simple Sequence Repeat
PCoA: Principal Coordinates Analysis
*R*_ST_: *R*-statistics
SM: Simple matching
SSRs: Simple sequence repeats
UPGMA: Unweighted pair group method using an Arithmetic average
Φ_PT_: PhiPT.
