# Supplementary material for: Genetic Biodiversity of Italian Olives (Olea europaea) Germplasm Analyzed by SSR Markers
Source: ScientificWorldJournal. 2014 Feb 27;2014:296590. doi: 10.1155/2014/296590 (PMC3958686; doi:10.1155/2014/296590)
Supplement: Supplementary file 1 — The electronic supplementary material of the research article “Genetic biodiversity of Italian olives (Olea europaea) germplasm analyzed by SSR markers” by Muzzalupo et al. is composed of three tables. Table S1: List of the Olea europaea L. varieties present in the Agricultural Research Council-Olive Growing and Oil Industry Research Centre (CRA-OLI) germplasm collection and analyzed by eleven microsatellite markers. The Italian olive varieties included in the core collection are indicated in bold. Table S2: Alleles detected at the eleven SSR loci in the 489 olive trees analyzed. For each allele, the size and the frequency are reported. Table S3: Genotype profiles obtained from the combination of the eleven microsatellite markers on 489 olive varieties. The Italian olive varieties included in the core collection are indicated in bold. [file 296590.f1.zip › Table S1.doc]

**Table S1 List of the *Olea europaea* L.varieties present in the CRA-OLI germplasm collection and analyzed by microsatellite markers. The Italian olive varieties included in the core collection are indicated in bold.**

| **N.** | **Varieties** | **Regions** | **Code** |
| --- | --- | --- | --- |
| 1 | Abunara | Sicily | B # 205 |
| 2 | Agristigna | Calabria | C1 # 319 |
| 3 | Aitana | Sicily | B # 206 |
| 4 | Albatro | Toscany | C1 # 270 |
| **5** | **Allora** | Toscany | C1 # 271 |
| 6 | Amati | Lazio | C1 # 338 |
| 7 | Americano | Toscany | A # 139 |
| 8 | Annarea | Basilicata | B # 262 |
| 9 | Arancino | Toscany | C1 # 272 |
| **10** | **Arnasca 1** | Liguria | A # 46A |
| 11 | Arnasca 2 | Liguria | A # 46B |
| 12 | Arnasca 3 | Liguria | A # 46C |
| **13** | **Arnasca 4** | Liguria | A # 46D |
| 14 | Arnasca 5 | Liguria | A # 46E |
| 15 | Arnasca 6 | Liguria | A # 46 |
| 16 | Ascolana dura | Marche | B # 229 |
| 17 | Ascolana semitenera | Marche | B # 230 |
| 18 | Ascolana tenera | Marche | A # 53 |
| 19 | Augellina | Basilicata | A # 10 |
| 20 | Aurina | Molise | B # 184 |
| 21 | Barilari | Sicily | C1 # 346 |
| 22 | Bianchella | Umbria | A # 128 |
| 23 | Bianchera | Friuli Venezia Giulia | A # 159 |
| 24 | Biancolella | Campania | C2 # 358 |
| 25 | Biancolilla | Sicily | C1 # 347 |
| 26 | Borgese | Calabria | A # 18 |
| 27 | Borgiona | Umbria | B # 180 |
| 28 | Bosana | Sardinia | A # 71 |
| 29 | Bottone di Gallo 1 | Sicily | C1 # 308A |
| 30 | Bottone di Gallo 2 | Sicily | C1 # 308B |
| 31 | Bottoni di Gallo | Molise | A # 174 |
| 32 | Brandofino | Sicily | A # 78 |
| 33 | Buga | Friuli Venezia Giulia | C1 # 337 |
| 34 | Buscionetto 1 | Sicily | A # 79A |
| 35 | Buscionetto 2 | Sicily | A # 79B |
| 36 | Buscionetto 3 | Sicily | A # A32 |
| 37 | Cacaredda | Sicily | B # 207 |
| 38 | Cacaridduni | Sicily | B # 208 |
| 39 | Cacazzara | Sicily | C2 # 383 |
| 40 | Caiazzana | Campania | B # 240 |
| 41 | Calatina | Sicily | A # 124 |
| 42 | Cammarotana | Campania | C1 # 324 |
| 43 | Canino 1 | Lazio | A # 32A |
| 44 | Canino 2 | Lazio | A # 32B |
| 45 | Capolga | Marche | B # 231 |
| **46** | **Caprina di Casalanguida** | Abruzzo | B # 260 |
| 47 | Caprina Vastese | Abruzzo | B # 261 |
| 48 | Carbona | Friuli Venezia Giulia | C2 # 367 |
| 49 | Carboncella | Lazio | A # 33 |
| 50 | Carboncella Campana | Campania | C2 # 359 |
| 51 | Carboncella Pianacce A | Lazio | B # A5 |
| 52 | Carboncella Pianacce B | Lazio | B # A6 |
| 53 | Carbonchio | Abruzzo | B # 216 |
| 54 | Carbuncion | Emilia Romagna | C1 # 334 |
| 55 | Carbuncion di Carpinenga | Emilia Romagna | C2 # 397 |
| 56 | Cariasina | Sardinia | A # 72 |
| 57 | Cariola | Calabria | C2 # 400 |
| 58 | Carmelitana | Apulia | A # 120 |
| 59 | Carolea Cefaly | Calabria | B # A7 |
| 60 | Carolea Cetraro | Calabria | B # A38 |
| 61 | Carolea Mirto | Calabria | A # 19 |
| 62 | Carolea Rende | Calabria | R P1F14 |
| 63 | Carolea Rossi | Calabria | B # A8 |
| 64 | Carpellese | Campania | C1 # 325 |
| 65 | Carpinella | Basilicata | C2 # 390 |
| **66** | **Carpinetana** | Abruzzo | B # 204 |
| 67 | Casaliva | Lombardy | A # 51 |
| 68 | Castiglionese | Abruzzo | A # 1 |
| 69 | Castricianella Rapparina | Sicily | B # 209 |
| 70 | Cavalieri | Sicily | B # 182 |
| 71 | Cazzarella | Molise | C2 # 369 |
| 72 | Cazzinicchio | Apulia | A # 121 |
| 73 | Cellacchia | Lazio | C1 # 339 |
| **74** | **Cellina di Nardo'** | Apulia | A # 55 |
| 75 | Cellina di Rotello | Molise | B # 185 |
| 76 | Cerasa di Montenero | Molise | C2 # 370 |
| 77 | Cerasuola | Sicily | A # 80 |
| 78 | Ciciarello | Calabria | A # 115 |
| 79 | Cicinella | Campania | C1 # 326 |
| 80 | Ciliegino | Toscany | C1 # 273 |
| 81 | Cima di Melfi | Basilicata | A # 11 |
| 82 | Cima di Mola | Apulia | A # 56 |
| 83 | Colombina | Emilia Romagna | B # 198 |
| 84 | Coratina 1 | Apulia | C2 # A2-057 |
| 85 | Coratina 2 | Apulia | A # A24 |
| 86 | Coratina 3 | Apulia | A # 57 |
| 87 | Cornacciola | Basilicata | C2 # 391 |
| 88 | Corneglia | Campania | B # 241 |
| 89 | Cornia | Campania | B # 242 |
| 90 | Corniola | Calabria | B # 192 |
| 91 | Corniolo | Umbria | A # 170 |
| 92 | Cornula | Apulia | R P1F08 |
| 93 | Coroncina | Marche | A # 103 |
| 94 | Correggiolo | Toscany | A # 126 |
| 95 | Correggiolo di Montegridolfo | Toscany | A # A30 |
| 96 | Correggiolo di Pallese | Toscany | C1 # 274 |
| 97 | Corsicana da mensa | Sardinia | C1 # 311 |
| 98 | Corsicana da olio | Sardinia | A # 73 |
| 99 | Craputea | Emilia Romagna | C1 # 264 |
| 100 | Crastu | Sicily | C1 # 348 |
| 101 | Crognolo | Lazio | C1 # 340 |
| 102 | CSM | Toscany | B # A3 |
| 103 | CSS-02-Minerva | Toscany | C1 # A355 |
| 104 | Cucca 1 | Toscany | A # 127 |
| 105 | Cucca 2 | Toscany | C1 # A1-127 |
| 106 | Curatora | Campania | C2 # 366 |
| 107 | Demoniata | Sicily | C2 # 384 |
| 108 | Dolce Agogia | Umbria | A # 97 |
| 109 | Dolce di Andria | Apulia | B # 196 |
| 110 | Dolce di Cassano | Apulia | A # 122 |
| 111 | Dolce di Cerchiara | Calabria | C1 # 320 |
| 112 | Dolce di Rossano | Calabria | A # 26 |
| **113** | **Don Carlo** | Umbria | C2 # 389 |
| 114 | Donna Francesca | Apulia | C2 # 403 |
| 115 | Dritta | Abruzzo | A # 4 |
| 116 | Dritta Loreto | Marche | B # 232 |
| 117 | Emilia | Toscany | C1 # 276 |
| 118 | Erbano 1 | Sicily | C1 # 349A |
| 119 | Erbano 2 | Sicily | C1 # 349B |
| 120 | Faresana | Basilicata | B # 202 |
| 121 | Faresana | Basilicata | C1 # A1-202 |
| 122 | Fasolina | Basilicata | C1 # 265 |
| 123 | Fasolona | Basilicata | C1 # 266 |
| 124 | Favarol | Veneto | A # 119 |
| 125 | Fecciaro | Umbria | A # 171 |
| 126 | Feglina | Liguria | A # 47 |
| 127 | Femminella di Torraca | Campania | B # 243 |
| 128 | Filare | Toscany | C1 # 277 |
| 129 | Fosco | Lazio | B # 227 |
| 130 | Frangivento | Apulia | A # 58 |
| 131 | Frantoio 1 | Toscany | C2 # A8-092 |
| 132 | Frantoio 10 | Toscany | B # A11 |
| 133 | Frantoio 2 | Toscany | A # 92 |
| 134 | Frantoio 3 | Toscany | C2 # A3-092 |
| 135 | Frantoio 4 | Toscany | C2 # A1-092 |
| 136 | Frantoio 5 | Toscany | A # A6-092 |
| 137 | Frantoio 6 | Toscany | B # A9 |
| 138 | Frantoio 7 | Toscany | B # A10A-092 |
| 139 | Frantoio 8 | Toscany | B # A10B-092 |
| 140 | Frantoio 9 | Toscany | C2 # A7-092 |
| 141 | Frantoio di Montegridolfo | Toscany | A # A28 |
| 142 | Frantoio di Villa Verrucchio | Toscany | A # A29 |
| 143 | Frantoio FC | Toscany | B # A12 |
| 144 | Fs17 | Umbria | A # 154 |
| 145 | Gaggiolo | Lombardy | A # 52 |
| **146** | **Gentile dell'Aquila** | Abruzzo | B # 217 |
| 147 | Gentile di Chieti | Abruzzo | A # 5 |
| 148 | Gentile di Larino 1 | Molise | A # 166A |
| 149 | Gentile di Larino 5 | Molise | A # 166B |
| 150 | Gentile nera di Colletorto | Molise | B # 190 |
| 151 | Ghiacciolo | Calabria | C2 # 398 |
| 152 | Ghiacciora | Calabria | R P1F10 |
| 153 | Ghiandaro | Abruzzo | A # 2 |
| 154 | Ghiannara | Basilicata | B # 263 |
| 155 | Giardino | Liguria | A # 121 |
| 156 | Giarfara | Sicily | B # 210 |
| 157 | Giarraffa 1 | Sicily | A # 81A |
| 158 | Giarraffa 2 | Sicily | A # 81B |
| 159 | Ginestrino | Toscany | C1 # 279 |
| 160 | Giusta | Basilicata | B # 223 |
| 161 | Gnagnaro | Molise | B # 187 |
| 162 | Grappolo | Toscany | C1 # 238 |
| 163 | Grappuda | Emilia Romagna | C2 # 388 |
| 164 | Gremignolo | Toscany | C1 # 281 |
| 165 | Gremignolo di Bolgheri | Toscany | C1 # 282 |
| 166 | Grignan | Veneto | A # 157 |
| 167 | Grossa di Cassano | Calabria | A # 20 |
| 168 | Grossa di Gerace | Calabria | A # 21 |
| 169 | Grossa di Spagna | Apulia | A # 59 |
| 170 | Grossa di Venafro | Molise | B # 188 |
| 171 | Grossale | Campania | B # 244 |
| 172 | I 77 | Umbria | B # 179 |
| 173 | Iacona 1 | Sicily | C1 # 350A |
| 174 | Iacona 2 | Sicily | C1 # 350B |
| **175** | **Imperiale** | Calabria | A # 158 |
| 176 | Intosso | Abruzzo | A # 6 |
| 177 | Itrana | Lazio | A # 34 |
| 178 | Larcianese | Toscany | C1 # 284 |
| **179** | **Lastrino** | Toscany | C1 # 285 |
| 180 | Laurina | Marche | A # 151 |
| **181** | **Lavagnina** | Liguria | A # 164 |
| 182 | Lazzera Reale | Toscany | C1 # 286 |
| 183 | Lea | Marche | A # 104 |
| 184 | Leccino 1 | Toscany | C2 #A9-093 |
| 185 | Leccino 10 | Toscany | B # A20-093 |
| 186 | Leccino 2 | Toscany | C1 # A8-093 |
| 187 | Leccino 3 | Toscany | C1 # A7-093 |
| 188 | Leccino 4 | Toscany | A # 93 |
| 189 | Leccino 5 | Toscany | A # A27 |
| 190 | Leccino 6 | Toscany | A # A6-093 |
| 191 | Leccino 7 | Toscany | A # A5-093 |
| 192 | Leccino 8 | Toscany | A # A4-093 |
| 193 | Leccino 9 | Toscany | A # A3-093 |
| 194 | Leccino Compatto | Toscany | B # A17 |
| 195 | Leccino Dwarf | Toscany | B # A18 |
| 196 | Leccino Minerva 1 | Toscany | A # A26A |
| 197 | Leccino Minerva 2 | Toscany | A # A26A |
| 198 | Leccino Pisa 4 | Toscany | C2 # 093A |
| 199 | Leccino Pisa 9 | Toscany | C2 # 093B |
| 200 | Leccio del Corno 1 | Toscany | C1 # A1-134 |
| 201 | Leccio del Corno 2 | Toscany | A # 134 |
| 202 | Leccio Marammano 1 | Toscany | C1 # 287 |
| 203 | Leccio Maremmano 2 | Toscany | C1 # 287 |
| 204 | Leccione | Toscany | C1 # 288 |
| 205 | Lezze | Apulia | A # 145 |
| 206 | Lumiaru | Sicily | B # 194 |
| 207 | Mafra | Calabria | A # 22 |
| 208 | Mafra di Cerchiara | Calabria | C1 # 321 |
| 209 | Maglianese | Marche | C2 # 368 |
| 210 | Maiatica di Ferrantina 1 | Basilicata | A # 12A |
| 211 | Maiatica di Ferrantina 2 | Basilicata | A # 12B |
| 212 | Majorca | Sardinia | C2 # 378 |
| 213 | Mandanici | Sicily | A # 125 |
| 214 | Manna | Sardinia | C2 # 379 |
| 215 | Mantonica | Sicily | B # 236 |
| 216 | Marchiaio | Toscany | B # 203 |
| 217 | Marina | Lazio | A # 35 |
| 218 | Marinese | Campania | C2 # 360 |
| 219 | Marzio | Toscany | A # 142 |
| 220 | Maurino 1 | Toscany | C2 #A4-135 |
| 221 | Maurino 2 | Toscany | C1 # A2-135 |
| 222 | Maurino 3 | Toscany | C1 # A3-135 |
| 223 | Maurino 4 | Toscany | A # 135 |
| 224 | Mele | Apulia | A # 60 |
| 225 | Mignola | Marche | A # 54 |
| 226 | Mignolo | Toscany | A # 136 |
| 227 | Mignolo Cerretano | Toscany | C1 # 290 |
| 228 | Minna di Vacca | Sicily | B # 211 |
| 229 | Minuta 1 | Sicily | A # 83A |
| 230 | Minuta 2 | Sicily | A # 83B |
| 231 | Minuta3 | Sicily | A # 83c |
| 232 | Minutella | Lazio | A # 36 |
| 233 | Monaca | Sicily | B # 212 |
| 234 | Mora | Apulia | A # 131 |
| 235 | Moraiolo 1 | Toscany | C2 #A5-094 |
| **236** | **Moraiolo 2** | Toscany | C1 # A3-094 |
| 237 | Moraiolo 3 | Toscany | C1 # A4-094 |
| 238 | Moraiolo 4 | Toscany | A # 94 |
| 239 | Moraiolo 5 | Toscany | B # A21 |
| 240 | Moraiolo T. Corsini | Toscany | A # 137 |
| 241 | Morcaio | Toscany | C1 # 291 |
| 242 | Morchiaio | Toscany | B # 203 |
| 243 | Morcone | Toscany | C1 # 293 |
| 244 | Morellona di Grecia | Apulia | B # 178 |
| 245 | Moresca 1 | Sicily | A # 84A |
| 246 | Moresca 2 | Sicily | A # 84B |
| 247 | Morinello | Toscany | B # 239 |
| 248 | Murtiddara | Sicily | C2 # 385 |
| 249 | Nasitana frutto grosso | Sicily | B # 195 |
| 250 | Nebba | Sicily | B # 259 |
| 251 | Nebbia | Marche | A # 105 |
| 252 | Nebbio di Chieti | Abruzzo | A # 102 |
| 253 | Nebbio di Pescara | Abruzzo | B # 176 |
| 254 | Negrera | Liguria | A # 152 |
| 255 | Nera di Cantinelle | Calabria | A # 23 |
| 256 | Nera di Gonnos | Sardinia | A # 74 |
| 257 | Nera di Oliena | Sardinia | C1 # 312 |
| 258 | Nera di Villacidro | Sardinia | A # 113 |
| 259 | Nerba | Sicily | B # 258 |
| 260 | Nerba Catanese | Sicily | C1 # 309 |
| 261 | Noccioluta | Molise | C2 # 371 |
| 262 | Nocellara del Belice 1 | Sicily | A # 85 |
| 263 | Nocellara del Belice 2 | Sicily | A # 85 |
| 264 | Nocellara etnea | Sicily | A # 86 |
| 265 | Nocellara etnea ovale | Sicily | B # 183 |
| 266 | Nocellara messinese | Sicily | A # 87 |
| 267 | Nocellara Nissena 1 | Sicily | C1 # 351A |
| 268 | Nocellara Nissena 2 | Sicily | C1 # 351B |
| 269 | Nociara | Apulia | A # 61 |
| 270 | Nolca | Apulia | A # 62 |
| 271 | Nostrale di Fiano | Lazio | A # 37 |
| 272 | Nostrale di Rigali | Umbria | A # 98 |
| 273 | Nostrana | Calabria | A # 24 |
| 274 | Nostrana Campana | Campania | B # 200 |
| 275 | Nostrana di Brisighella 1 | Emilia Romagna | A # 148A |
| 276 | Nostrana di Brisighella 2 | Emilia Romagna | A # 148B |
| 277 | Nostrana di Brisighella 3 | Emilia Romagna | A # 148C |
| 278 | Nostrana di Brisighella 4 | Emilia Romagna | A # A31 |
| 279 | Ogliara | Campania | B # 245 |
| 280 | Ogliarola Barese | Apulia | A # 63 |
| 281 | Ogliarola del Bradano 1 | Basilicata | C2 # A1-013 |
| 282 | Ogliarola del Bradano 2 | Basilicata | A # 13 |
| 283 | Ogliarola del Murge | Apulia | R P1F09 |
| 284 | Ogliarola di Montalbano | Basilicata | A # 14 |
| 285 | Ogliarola Garganica | Apulia | B # 257 |
| 286 | Ogliarola Messinese 1 | Sicily | A # 88A |
| 287 | Ogliarola Messinese 2 | Sicily | A # 88B |
| 288 | Ogliarola Salentina | Apulia | A # 64 |
| 289 | OgliarolaVulture | Basilicata | A # 15 |
| 290 | Ogliastro grande | Campania | B # 246 |
| 291 | Olianedda | Sardinia | C2 # 380 |
| 292 | Oliastro | Apulia | A # 112 |
| 293 | Olieddu | Sardinia | C1 # 313 |
| 294 | Oliva bianca | Campania | C2 # 361 |
| 295 | Oliva da polpa | Campania | C2 # 365 |
| 296 | Oliva grossa | Emilia Romagna | B # 199 |
| 297 | Oliva Rossa | Apulia | C2 # 376 |
| 298 | Oliva torsa | Campania | C2 # 362 |
| 299 | Oliva vera | Umbria | C1 # 357 |
| 300 | Olivago | Umbria | A # 155 |
| 301 | Olivastra di Populonia | Toscany | C1 # 294 |
| 302 | Olivastra Seggianese | Toscany | A # 173 |
| **303** | **Olivastro d'Aprile** | Molise | C2 # 372 |
| 304 | Olivastro di Bucchianico | Abruzzo | B # 218 |
| **305** | **Olivastro di Montenero** | Molise | C2 # 374 |
| 306 | Olivastro Dritto | Molise | C2 # 373 |
| 307 | Olivastro Frentano | Abruzzo | B # 219 |
| 308 | Olivella appuntita | Campania | C1 # 327 |
| 309 | Olivella di Cerchiara | Calabria | C1 # 322 |
| 310 | Olivella di Grottaminarda | Campania | C2 # 363 |
| 311 | Olivetta Nera | Molise | C2 # 375 |
| 312 | Olivo da olio | Campania | B # 247 |
| 313 | Olivo da salare | Campania | B # 248 |
| 314 | Olivo del mulino | Toscany | A # 140 |
| 315 | Olivo della Madonna | Toscany | A # 141 |
| 316 | Olivo delle Alpi | Liguria | C1 # 342 |
| 317 | Olivo di Casavecchia | Toscany | C1 # 295 |
| 318 | Olivo di Castiglione | Sicily | C1 # 310 |
| 319 | Olivo di San Lorenzo | Toscany | C1 # 296 |
| 320 | Olivoce | Abruzzo | A # 3 |
| 321 | Olivomensa | Basilicata | C1 # 267 |
| 322 | Olivona | Apulia | R P1F03 |
| 323 | Olivone di Viterbo | Lazio | C1 # 341 |
| 324 | OperaPia | Sicily | R P1F06 |
| 325 | Orazio | Basilicata | C2 # 392 |
| 326 | Orbetana | Marche | A # 107 |
| 327 | Ornellaia | Toscany | C1 # 297 |
| 328 | Ortice | Campania | B # 201 |
| 329 | Ortolana | Campania | A # 161 |
| 330 | Ottobratica | Calabria | A # 25 |
| 331 | Ottobrina | Liguria | A # 48 |
| 332 | Paesana Bianca | Molise | B # 233 |
| 333 | Paesana Nera | Molise | B # 234 |
| 334 | Palma | Sardinia | C2 # 381 |
| 335 | Palmarola | Basilicata | C1 # 268 |
| 336 | Pampagliosa | Campania | A # 162 |
| 337 | Paschixedda | Sardinia | C1 # 314 |
| 338 | Pasola | Apulia | A # 65 |
| 339 | Passulunara | Sicily | A # 143 I |
| 340 | Patrinostraru | Sicily | C2 # 386 |
| 341 | Pendolino 1 | Toscany | C2 # A4-095 |
| 342 | Pendolino 2 | Toscany | C1 # A3-095 |
| 343 | Pendolino 3 | Toscany | C1 # A2-095 |
| 344 | Pendolino 4 | Toscany | C1 # A1-095 |
| 345 | Pendolino 5 | Toscany | A # 95 |
| 346 | Pennulara | Calabria | B # 225 |
| 347 | Peppino Leo | Apulia | A # 132 |
| 348 | Peranzana | Apulia | A # 66 |
| 349 | Perciasacchi | Campania | B # 249 |
| 350 | Pescarese | Abruzzo | A # 7 |
| 351 | Pesciatino | Toscany | C1 # 298 |
| **352** | **Pezz'e quaddu** | Sardinia | C2 # 382 |
| 353 | Piangente 1 | Toscany | C1 # A1-138 |
| 354 | Piangente 2 | Toscany | A # 138A |
| 355 | Piangente 3 | Toscany | A # 138B |
| 356 | Piantone di Falerone | Marche | A # 106 |
| 357 | Piantone di Macerata | Marche | C2 # 399 |
| 358 | Piantone di Mogliano | Marche | A # 108 |
| 359 | Pidicuddara | Sicily | A # 153 |
| 360 | Pignola | Liguria | A # 150 |
| 361 | Pirunara 1 | Sicily | C1 # 352A |
| 362 | Pirunara 2 | Sicily | C1 # 352B |
| 363 | Pisciottana | Campania | A # 31 |
| 364 | Pizz'e Carroga | Sardinia | A # 75 |
| 365 | Pizzo di corvo 1 | Sicily | C1 # 353A |
| 366 | Pizzo di corvo 2 | Sicily | C1 # 353B |
| 367 | Pizzutella | Sicily | B # 213 |
| 368 | Pocciolo | Umbria | C1 # 356 |
| 369 | Posola | Abruzzo | B # 220 |
| 370 | Posolella | Abruzzo | B # 221 |
| 371 | Precoce | Abruzzo | A # 146 |
| 372 | Procanica | Lazio | A # 38 |
| 373 | Provenzale | Campania | B # 255 |
| 374 | Puntella | Abruzzo | B # 177 |
| 375 | Punteruolo | Toscany | C1 # 299 |
| 376 | Puntino | Toscany | C1 # 300 |
| 377 | Racemo 1 | Apulia | C1 # 344 |
| 378 | Racemo 2 | Apulia | C1 # 344 |
| 379 | Racioppa | Basilicata | C1 # 317 |
| 380 | Racioppa | Campania | B # 250 |
| 381 | Racioppella | Campania | A # 163 |
| **382** | **Raggiola** | Marche | A # 109 |
| 383 | Raja | Umbria | A # 99 |
| 384 | Raja Sabina | Lazio | A # 149 |
| 385 | Rajo | Umbria | A # 114 |
| 386 | Rastellina | Umbria | A # 172 |
| **387** | **Ravece** | Campania | C1 # 328 |
| 388 | Raza | Veneto | A # 156 |
| 389 | Razzaio | Toscany | C1 # 301 |
| 390 | Razzo 1 | Toscany | C2 # A1-169 |
| 391 | Razzo 2 | Toscany | A # 169 |
| 392 | Razzola | Liguria | A # 49 |
| 393 | Reale | Lazio | A # 39 |
| 394 | Regina | Apulia | R P1F07 |
| 395 | Resciola di Venafro | Molise | B # 189 |
| 396 | Riminino | Lazio | A # 40 |
| **397** | **Ritonnella** | Campania | B # 251 |
| 398 | Rizza | Basilicata | A # 16 |
| 399 | Rizzitella | Campania | B # 252 |
| 400 | Roma | Basilicata | C2 # 393 |
| 401 | Romanella | Basilicata | C2 # 394 |
| 402 | Romanella | Calabria | A # 129 |
| 403 | Romanella Molisana | Molise | A # 167 |
| 404 | Rosciola | Marche | A # 110 |
| 405 | Rosciola Coltodino | Lazio | B # 228 |
| **406** | **Rosciola di Rotello** | Molise | B # 186 |
| 407 | Rosciola di Venafro | Molise | B # 189 |
| 408 | Rosciola laziale | Lazio | A # 41 |
| 409 | Rosino | Toscany | C1 # 302 |
| 410 | Rossellino | Toscany | C1 # 303 |
| 411 | Rossello | Toscany | C1 # 304 |
| 412 | Rossina | Emilia Romagna | C1 # 335 |
| 413 | Rotondella | Basilicata | C1 # 296 |
| 414 | Rotondella di Foggia | Apulia | C2 # 377 |
| 415 | Rotondella di Sanza 1 | Campania | C1 # 329A |
| 416 | Rotondella di Sanza 2 | Campania | C1 # 329B |
| **417** | **Rumignana** | Molise | B # 191 |
| **418** | **Rustica** | Abruzzo | B # 222 |
| 419 | Ruveia | Campania | C1 # 330 |
| 420 | Salegna di Larino | Molise | B # 235 |
| 421 | Salella | Campania | C2 # 364 |
| 422 | Salicino | Toscany | C1 # 306 |
| 423 | Salvia | Lazio | A # 42 |
| 424 | Salviana | Lazio | A # 43 |
| 425 | Sammartinara | Sicily | B # 214 |
| 426 | Sammartinenga | Basilicata | B # 224 |
| 427 | San Benedetto | Apulia | B # 197 |
| 428 | San Felice Acquasparta 1 | Umbria | A # 100A |
| 429 | San Felice Acquasparta 2 | Umbria | A # 100B |
| 430 | San Felice Acquasparta 3 | Umbria | A # 100C |
| 431 | San Felice Acquasparta 4 | Umbria | A # 100D |
| 432 | San Felice Acquasparta 5 | Umbria | A # 100E |
| 433 | San Francesco | Toscany | A # 144 |
| 434 | San Lazzero | Toscany | C1 # 305 |
| 435 | Sanginara | Campania | C1 # 331 |
| **436** | **Santa Caterina** | Toscany | A # 96 |
| 437 | Santa Maria | Campania | B # 253 |
| 438 | Sant'Agatese | Sicily | A # 89 |
| 439 | Sant'Agostino | Apulia | A # 67 |
| 440 | Santomauro | Calabria | A # 160 |
| 441 | Sargano | Marche | A # 111 |
| 442 | Sargano | Marche | A # 165 |
| 443 | Scarpetta | Basilicata | C1 # 318 |
| 444 | Selvatico | Emilia Romagna | C1 # 336 |
| 445 | Semidana | Sardinia | A # 76 |
| 446 | Sessana | Campania | B # 256 |
| 447 | Simona | Apulia | A # 68 |
| 448 | Sinopolese | Calabria | A # 27 |
| 449 | Sirole | Lazio | A # 44 |
| 450 | Sivigliana da olio | Sardinia | C1 # 345 |
| 451 | Spagnola di Missano | Liguria | C1 # 343 |
| 452 | Sperone di Gallo | Molise | A # 175 |
| 453 | Spezzanese | Calabria | C1 # 323 |
| 454 | Spinoso | Basilicata | C2 # 395 |
| 455 | Taggiasca | Liguria | A # 50 |
| 456 | Tenacella | Campania | C1 # 332 |
| 457 | Tendellone | Umbria | A # 101 |
| 458 | Termite di Bitetto | Apulia | A # 69 |
| 459 | Terza grande | Sardinia | A # 133 |
| 460 | Terza piccola | Sardinia | C1 # 316 |
| **461** | **Toccolana** | Abruzzo | A # 8 |
| 462 | Tombarello | Calabria | B # 193 |
| 463 | Tonda di Alife | Campania | B # 254 |
| 464 | Tonda di Cagliari | Sardinia | A # 77 |
| 465 | Tonda di Filadelfia | Calabria | A # 28 |
| 466 | Tonda di Filogaso | Calabria | A # 29 |
| 467 | Tonda di Strongoli | Calabria | A # 116 |
| 468 | Tonda Dolce | Calabria | B # 226 |
| 469 | Tonda Dolce di Partanna | Sicily | B # 237 |
| 470 | Tonda iblea | Sicily | A # 90 |
| 471 | Tondina | Calabria | A # 30 |
| 472 | Tortiglione | Abruzzo | A # 9 |
| 473 | Toscanina | Apulia | A # 70 |
| 474 | Tunnulidda | Sicily | B # 215 |
| 475 | Turdunazza antimosca 1 | Sicily | C2 # 387A |
| 476 | Turdunazza antimosca 2 | Sicily | C2 # 387B |
| 477 | Uccellara | Basilicata | A # 17 |
| 478 | Ugea di Savoia | Apulia | R P1F05 |
| 479 | Ugliastride | Apulia | R P1F04 |
| 480 | Vaddara | Sicily | C1 # 354 |
| 481 | Vallanella 1 | Lazio | A # 45A |
| 482 | Vallanella 2 | Lazio | A # 45B |
| 483 | Verdello | Sicily | A # 91 |
| 484 | Vigna della Corte | Campania | C1 # 333 |
| 485 | Vocio | Umbria | B # 181 |
| 486 | Vraja | Calabria | C2 # 396 |
| 487 | Zaituna | Sicily | A # 82A |
| 488 | Zimbimbo | Apulia | A # 123 |
| 489 | Zinzifarica | Calabria | A # 147 |
