# Supplementary material for: Genetic Biodiversity of Italian Olives (Olea europaea) Germplasm Analyzed by SSR Markers
Source: ScientificWorldJournal. 2014 Feb 27;2014:296590. doi: 10.1155/2014/296590 (PMC3958686; doi:10.1155/2014/296590)
Supplement: Supplementary file 1 — The electronic supplementary material of the research article “Genetic biodiversity of Italian olives (Olea europaea) germplasm analyzed by SSR markers” by Muzzalupo et al. is composed of three tables. Table S1: List of the Olea europaea L. varieties present in the Agricultural Research Council-Olive Growing and Oil Industry Research Centre (CRA-OLI) germplasm collection and analyzed by eleven microsatellite markers. The Italian olive varieties included in the core collection are indicated in bold. Table S2: Alleles detected at the eleven SSR loci in the 489 olive trees analyzed. For each allele, the size and the frequency are reported. Table S3: Genotype profiles obtained from the combination of the eleven microsatellite markers on 489 olive varieties. The Italian olive varieties included in the core collection are indicated in bold. [file 296590.f1.zip › Table S2.doc]

**Table S2** Alleles detected at the SSR loci in the 489 olive trees analyzed. For each allele, the size (expressed in base pairs) and the frequency are reported.

| Locus | **Alleles (bp)** | | | | | | | | | | | |
| --- | --- | --- | --- | --- | --- | --- | --- | --- | --- | --- | --- | --- |
|  | **1** | **2** | **3** | **4** | **5** | **6** | **7** | **8** | **9** | **10** | **11** | **12** |
| GAPU59 | 208 | 212 | 214 | 218 | 222 |  |  |  |  |  |  |  |
| *Frequency* | *0.165* | *0.331* | *0.237* | *0.069* | *0.198* |  |  |  |  |  |  |  |
| GAPU71A | 210 | 212 | 214 | 218 | 221 | 224 | 228 | 250 | 259 |  |  |  |
| *Frequency* | *0.072* | *0.069* | *0.485* | *0.137* | *0.037* | *0.192* | *0.001* | *0.002* | *0.005* |  |  |  |
| GAPU71B | 121 | 124 | 126 | 130 | 144 |  |  |  |  |  |  |  |
| *Frequency* | *0.012* | *0.253* | *0.227* | *0.163* | *0.345* |  |  |  |  |  |  |  |
| GAPU103A | 136 | 150 | 157 | 159 | 170 | 184 | 197 | 203 |  |  |  |  |
| *Frequency* | *0.089* | *0.132* | *0.188* | *0.233* | *0.186* | *0.146* | *0.010* | *0.016* |  |  |  |  |
| UDO01 | 140 | 144 | 150 |  |  |  |  |  |  |  |  |  |
| *Frequency* | *0.304* | *0.451* | *0.246* |  |  |  |  |  |  |  |  |  |
| UDO03 | 135 | 143 | 150 | 166 | 182 | 202 |  |  |  |  |  |  |
| *Frequency* | *0.191* | *0.429* | *0.306* | *0.031* | *0.031* | *0.012* |  |  |  |  |  |  |
| UDO12 | 156 | 164 | 166 | 177 | 182 | 193 |  |  |  |  |  |  |
| *Frequency* | *0.005* | *0.037* | *0.326* | *0.290* | *0.100* | *0.242* |  |  |  |  |  |  |
| UDO28 | 143 | 150 | 154 | 156 | 161 | 182 | 205 | 210 | 245 |  |  |  |
| *Frequency* | *0.114* | *0.063* | *0.148* | *0.005* | *0.077* | *0.275* | *0.183* | *0.133* | *0.003* |  |  |  |
| UDO39 | 108 | 125 | 164 | 170 | 185 | 200 | 205 | 209 | 213 | 220 | 232 | 243 |
| *Frequency* | *0.028* | *0.014* | *0.017* | *0.025* | *0.145* | *0.110* | *0.269* | *0.009* | *0.229* | *0.075* | *0.070* | *0.010* |
| DCA09 | 162 | 166 | 172 | 176 | 182 | 184 | 188 | 194 | 198 | 204 | 206 | 210 |
| *Frequency* | *0.115* | *0.006* | *0.137* | *0.068* | *0.187* | *0.002* | *0.003* | *0.081* | *0.211* | *0.005* | *0.174* | *0.013* |
| DCA18 | 163 | 169 | 173 | 175 | 177 | 179 | 181 | 183 | 185 |  |  |  |
| *Frequency* | *0.06* | *0.013* | *0.023* | *0.012* | *0.194* | *0.312* | *0.300* | *0.003* | *0.137* |  |  |  |
