# Supplementary material for: Genetic Biodiversity of Italian Olives (Olea europaea) Germplasm Analyzed by SSR Markers
Source: ScientificWorldJournal. 2014 Feb 27;2014:296590. doi: 10.1155/2014/296590 (PMC3958686; doi:10.1155/2014/296590)
Supplement: Supplementary file 1 — The electronic supplementary material of the research article “Genetic biodiversity of Italian olives (Olea europaea) germplasm analyzed by SSR markers” by Muzzalupo et al. is composed of three tables. Table S1: List of the Olea europaea L. varieties present in the Agricultural Research Council-Olive Growing and Oil Industry Research Centre (CRA-OLI) germplasm collection and analyzed by eleven microsatellite markers. The Italian olive varieties included in the core collection are indicated in bold. Table S2: Alleles detected at the eleven SSR loci in the 489 olive trees analyzed. For each allele, the size and the frequency are reported. Table S3: Genotype profiles obtained from the combination of the eleven microsatellite markers on 489 olive varieties. The Italian olive varieties included in the core collection are indicated in bold. [file 296590.f1.zip › Table S3.doc]

**Table S3 Genotype profiles obtained from the combination of the eleven microsatellite markers on 489 olive varieties. The Italian olive varieties included in the core collection are indicated in bold.**

| **Varieties** | **GAPU59** | **GAPU71A** | **GAPU71B** | **GAPU103A** | **UDO001** | **UDO003** | **UDO012** | **UDO028** | **UDO039** | **DCA09** | **DCA18** |
| --- | --- | --- | --- | --- | --- | --- | --- | --- | --- | --- | --- |
| Abunara | 208-212 | 210-210 | 124-144 | 159-170 | 144-144 | 143-143 | 177-177 | 182-182 | 108-108 | 162-162 | 179-181 |
| Agristigna | 208-208 | 210-210 | 124-144 | 170-184 | 144-144 | 143-143 | 166-177 | 143-205 | 200-200 | 198-206 | 179-181 |
| Aitana | 208-212 | 210-210 | 124-144 | 150-184 | 144-144 | 143-143 | 166-193 | 182-182 | 108-108 | 176-176 | 179-179 |
| Albatro | 208-222 | 214-214 | 124-144 | 159-170 | 144-144 | 150-150 | 177-177 | 143-182 | 205-205 | 176-206 | 179-181 |
| **Allora** | **212-212** | **214-214** | **126-144** | **159-159** | **150-150** | **143-143** | **164-164** | **154-182** | **164-205** | **206-206** | **181-185** |
| Amati | 214-218 | 214-224 | 124-144 | 157-159 | 144-144 | 150-150 | 166-177 | 150-161 | 213-213 | 182-198 | 177-179 |
| Americano | 212-218 | 218-224 | 126-126 | 159-170 | 144-144 | 150-150 | 177-193 | 182-182 | 205-205 | 162-198 | 179-181 |
| Annarea | 212-222 | 214-224 | 124-144 | 184-184 | 144-144 | 150-150 | 166-193 | 182-210 | 205-205 | 182-182 | 179-181 |
| Arancino | 212-218 | 214-214 | 124-130 | 184-184 | 140-140 | 150-150 | 156-193 | 154-205 | 220-243 | 172-194 | 179-185 |
| **Arnasca 1** | **212-212** | **224-224** | **126-144** | **159-184** | **140-140** | **150-150** | **166-193** | **154-205** | **213-232** | **182-206** | **181-185** |
| Arnasca 2 | 212-212 | 224-224 | 126-144 | 159-184 | 140-140 | 150-150 | 166-193 | 154-205 | 213-232 | 182-206 | 181-185 |
| Arnasca 3 | 212-212 | 224-224 | 126-144 | 159-184 | 140-140 | 150-150 | 166-193 | 154-205 | 213-232 | 182-206 | 181-185 |
| **Arnasca 4** | **208-212** | **214-259** | **124-144** | **157-157** | **144-144** | **150-150** | **166-193** | **154-154** | **213-213** | **182-206** | **181-185** |
| Arnasca 5 | 212-212 | 224-224 | 126-144 | 159-184 | 140-140 | 150-150 | 166-193 | 154-205 | 213-232 | 182-206 | 181-185 |
| Arnasca 6 | 212-212 | 214-218 | 130-144 | 170-184 | 140-140 | 135-135 | 164-177 | 161-205 | 213-232 | 162-176 | 177-179 |
| Ascolana dura | 214-222 | 214-224 | 124-130 | 136-157 | 140-140 | 143-143 | 166-166 | 182-210 | 200-200 | 198-206 | 177-181 |
| Ascolana semitenera | 214-222 | 214-224 | 124-130 | 136-157 | 140-140 | 135-135 | 166-166 | 182-210 | 200-232 | 198-206 | 177-181 |
| Ascolana tenera | 214-222 | 214-224 | 126-144 | 136-184 | 140-140 | 143-143 | 166-166 | 150-161 | 200-200 | 198-206 | 177-179 |
| Augellina | 208-208 | 210-210 | 124-126 | 157-203 | 140-140 | 143-143 | 164-166 | 150-150 | 205-205 | 194-198 | 177-181 |
| Aurina | 222-222 | 210-214 | 124-144 | 159-170 | 144-144 | 150-150 | 177-177 | 154-205 | 213-213 | 162-206 | 173-175 |
| Barilari | 212-212 | 214-214 | 124-130 | 159-184 | 144-144 | 150-150 | 166-193 | 154-182 | 205-205 | 172-198 | 177-179 |
| Bianchella | 212-222 | 218-224 | 130-144 | 157-157 | 140-140 | 135-135 | 177-182 | 182-210 | 185-220 | 176-206 | 181-185 |
| Bianchera | 214-214 | 214-224 | 130-130 | 136-157 | 140-140 | 150-150 | 166-193 | 182-205 | 213-232 | 182-182 | 177-185 |
| Biancolella | 212-214 | 214-224 | 124-144 | 159-170 | 144-150 | 150-150 | 164-177 | 182-210 | 185-185 | 182-206 | 181-185 |
| Biancolilla | 212-212 | 210-214 | 121-130 | 136-150 | 144-144 | 150-150 | 166-193 | 143-205 | 108-108 | 172-198 | 177-179 |
| Borgese | 212-222 | 214-214 | 130-144 | 150-184 | 144-144 | 143-143 | 166-193 | 143-205 | 200-220 | 162-198 | 179-179 |
| Borgiona | 214-222 | 214-224 | 130-144 | 157-157 | 140-140 | 143-143 | 166-193 | 150-210 | 200-200 | 162-198 | 177-181 |
| Bosana | 214-222 | 214-218 | 130-144 | 170-184 | 144-150 | 135-135 | 177-193 | 161-205 | 205-205 | 194-198 | 181-185 |
| Bottone di Gallo 1 | 212 -222 | 214-259 | 124-126 | 150-184 | 144-144 | 150-150 | 177-193 | 154-182 | 220-220 | 162-172 | 179-185 |
| Bottone di Gallo 2 | 212 -222 | 214-259 | 124-126 | 150-184 | 144-144 | 150-150 | 177-193 | 154-182 | 220-220 | 162-182 | 179-185 |
| Bottoni di Gallo | 212-212 | 210-214 | 124-144 | 170-170 | 144-144 | 150-150 | 177-193 | 154-205 | 213-213 | 172-206 | 173-177 |
| Brandofino | 208-212 | 210-214 | 126-144 | 136-159 | 144-144 | 150-150 | 166-193 | 143-143 | 213-232 | 176-206 | 179-179 |
| Buga | 214-214 | 218-218 | 126-144 | 136-157 | 140-140 | 135-135 | 166-193 | 182-205 | 200-200 | 182-198 | 179-185 |
| Buscionetto 1 | 212 -222 | 210-214 | 124-130 | 159-184 | 144-144 | 150-150 | 166-166 | 154-154 | 108-213 | 176-198 | 179-181 |
| Buscionetto 2 | 212-212 | 210-214 | 124-126 | 136-150 | 140-140 | 150-150 | 166-193 | 154-154 | 213-213 | 176-198 | 181-181 |
| Buscionetto 3 | 212-212 | 210-214 | 124-126 | 136-150 | 140-140 | 150-150 | 166-193 | 154-154 | 213-213 | 176-198 | 181-181 |
| Cacaredda | 208-212 | 210-210 | 124-144 | 159-184 | 144-144 | 143-143 | 166-193 | 182-182 | 205-205 | 176-206 | 181-181 |
| Cacaridduni | 208-212 | 210-210 | 124-144 | 150-184 | 144-144 | 143-143 | 166-193 | 182-182 | 108-108 | 162-176 | 169-181 |
| Cacazzara | 208-212 | 214-214 | 124-126 | 157-184 | 144-144 | 150-150 | 166-193 | 182-182 | 185-185 | 176-176 | 179-179 |
| Caiazzana | 218-222 | 214-214 | 126-126 | 170-184 | 140-140 | 135-135 | 164-177 | 143-143 | 200-213 | 198-198 | 179-181 |
| Calatina | 212-222 | 210-214 | 124-126 | 136-136 | 144-144 | 150-150 | 177-177 | 143-182 | 205-205 | 182-182 | 175-179 |
| Cammarotana | 214-222 | 212-218 | 124-144 | 159-170 | 140-140 | 143-143 | 164-177 | 143-143 | 213-213 | 194-206 | 179-181 |
| Canino 1 | 212 -222 | 214-221 | 126-144 | 159-159 | 150-150 | 135-135 | 166-182 | 143-154 | 213-232 | 172-182 | 173-179 |
| Canino 2 | 212 -222 | 214-221 | 126-144 | 159-159 | 150-150 | 135-135 | 166-182 | 143-154 | 213-232 | 172-182 | 173-179 |
| Capolga | 214-214 | 214-224 | 124-144 | 136-150 | 144-144 | 150-150 | 166-193 | 154-205 | 220-232 | 176-198 | 181-185 |
| **Caprina di Casalanguida** | **208-212** | **214-214** | **124-124** | **150-150** | **144-144** | **150-182** | **166-193** | **154-205** | **213-213** | **162-204** | **177-177** |
| Caprina Vastese | 208-212 | 214-214 | 124-130 | 150-150 | 144-144 | 150-202 | 177-193 | 154-205 | 213-213 | 162-204 | 177-177 |
| Carbona | 214-214 | 214-224 | 126-130 | 150-157 | 150-150 | 143-143 | 166-193 | 143-210 | 185-185 | 176-198 | 179-181 |
| Carboncella | 208-212 | 214-224 | 124-144 | 170-184 | 144-144 | 150-150 | 166-193 | 182-210 | 213-213 | 162-194 | 177-181 |
| Carboncella Campana | 214-218 | 218-218 | 124-126 | 150-157 | 144-150 | 150-150 | 177-177 | 150-182 | 200-200 | 182-206 | 181-185 |
| Carboncella Pianacce A | 208-212 | 214-214 | 124-144 | 170-184 | 144-144 | 135-182 | 166-166 | 143-210 | 205-205 | 162-194 | 177-181 |
| Carboncella Pianacce B | 208-212 | 214-214 | 124-130 | 157-157 | 144-144 | 166-166 | 177-193 | 154-205 | 213-213 | 162-194 | 177-181 |
| Carbonchio | 212-218 | 214-214 | 124-124 | 150-170 | 140-140 | 150-150 | 177-193 | 154-205 | 213-213 | 198-210 | 181-185 |
| Carbuncion | 218-222 | 218-218 | 126-144 | 159-159 | 150-150 | 150-150 | 166-182 | 182-205 | 125-125 | 176-182 | 177-181 |
| Carbuncion di Carpinenga | 214-218 | 214-214 | 126-144 | 157-159 | 140-140 | 135-135 | 177-182 | 182-182 | 125-200 | 172-198 | 177-181 |
| Cariasina | 214-214 | 214-218 | 130-144 | 136-184 | 144-150 | 143-143 | 166-193 | 150-161 | 205-205 | 162-172 | 179-181 |
| Cariola | 218-222 | 212-212 | 130-144 | 184-184 | 144-144 | 150-150 | 166-193 | 143-154 | 205-205 | 162-198 | 181-185 |
| Carmelitana | 208-208 | 214-224 | 124-126 | 136-159 | 144-144 | 143-143 | 166-193 | 182-182 | 185-185 | 198-198 | 179-181 |
| Carolea Cefaly | 212-222 | 214-214 | 121-130 | 136-170 | 144-144 | 143-143 | 166-193 | 154-205 | 200-200 | 162-198 | 179-185 |
| Carolea Cetraro | 212-222 | 214-214 | 130-144 | 136-170 | 144-144 | 143-143 | 166-193 | 143-205 | 200-200 | 162-198 | 179-185 |
| Carolea Mirto | 212-222 | 214-214 | 121-130 | 136-170 | 144-144 | 143-143 | 166-193 | 154-205 | 200-200 | 162-198 | 179-185 |
| Carolea Rende | 212-222 | 214-214 | 121-130 | 136-184 | 144-144 | 143-143 | 166-193 | 154-205 | 125-213 | 162-198 | 179-181 |
| Carolea Rossi | 212-222 | 214-214 | 130-144 | 136-170 | 144-144 | 143-143 | 166-193 | 143-205 | 200-200 | 162-198 | 179-185 |
| Carpellese | 214-222 | 214-224 | 124-144 | 157-159 | 144-144 | 143-143 | 166-177 | 161-210 | 200-200 | 172-198 | 181-185 |
| Carpinella | 212-218 | 214-214 | 126-144 | 150-159 | 150-150 | 150-150 | 166-177 | 156-205 | 205-205 | 182-182 | 181-181 |
| **Carpinetana** | **212-222** | **214-214** | **126-144** | **184-184** | **144-144** | **150-150** | **177-193** | **154-210** | **205-205** | **166-206** | **177-181** |
| Casaliva | 208-212 | 214-224 | 124-144 | 159-170 | 144-144 | 143-143 | 177-182 | 182-205 | 205-205 | 172-194 | 179-181 |
| Castiglionese | 208-212 | 210-214 | 126-126 | 159-184 | 144-144 | 150-150 | 166-193 | 154-210 | 205-232 | 198-206 | 163-181 |
| Castricianella Rapparina | 208-222 | 210-214 | 124-130 | 159-170 | 144-144 | 150-150 | 177-177 | 154-205 | 170-213 | 198-206 | 179-179 |
| Cavalieri | 212-222 | 210-210 | 124-144 | 159-159 | 144-144 | 150-150 | 177-193 | 154-154 | 108-108 | 162-162 | 177-185 |
| Cazzarella | 212-212 | 214-214 | 121-121 | 170-170 | 140-140 | 143-143 | 166-193 | 182-182 | 213-213 | 162-172 | 169-179 |
| Cazzinicchio | 212-212 | 210-214 | 124-126 | 159-159 | 140-140 | 143-143 | 166-193 | 143-205 | 108-205 | 182-198 | 181-181 |
| Cellacchia | 212-218 | 218-224 | 124-144 | 150-184 | 144-144 | 135-135 | 166-166 | 143-143 | 205-205 | 182-198 | 181-181 |
| **Cellina di Nardo'** | **208-222** | **214-228** | **124-126** | **170-170** | **144-144** | **150-150** | **166-166** | **182-205** | **108-213** | **176-198** | **177-177** |
| Cellina di Rotello | 222-222 | 210-214 | 124-126 | 150-184 | 140-140 | 202-202 | 177-193 | 154-210 | 170-213 | 194-206 | 177-181 |
| Cerasa di Montenero | 212-222 | 214-214 | 126-144 | 170-184 | 144-144 | 143-143 | 177-193 | 143-182 | 205-205 | 176-206 | 173-179 |
| Cerasuola | 208-212 | 210-214 | 124-144 | 150-184 | 144-144 | 150-150 | 166-166 | 154-154 | 213-213 | 176-206 | 177-181 |
| Ciciarello | 212-212 | 212-212 | 124-124 | 159-170 | 144-144 | 143-143 | 177-193 | 143-143 | 205-205 | 198-198 | 177-177 |
| Cicinella | 214-218 | 212-218 | 124-130 | 170-184 | 140-140 | 143-143 | 166-193 | 154-161 | 205-205 | 172-198 | 185-185 |
| Ciliegino | 208-208 | 214-218 | 126-144 | 170-184 | 144-144 | 202-202 | 156-166 | 156-205 | 220-232 | 172-194 | 179-185 |
| Cima di Melfi | 212-222 | 210-224 | 124-144 | 170-170 | 144-144 | 150-150 | 166-193 | 154-210 | 170-213 | 194-194 | 181-181 |
| Cima di Mola | 212-212 | 210-214 | 124-144 | 159-159 | 144-144 | 143-143 | 177-193 | 150-182 | 170-170 | 162-162 | 173-177 |
| Colombina | 212-222 | 218-224 | 144-144 | 184-203 | 150-150 | 143-143 | 166-193 | 182-182 | 213-213 | 172-206 | 179-179 |
| Coratina 1 | 212-212 | 210-224 | 124-144 | 136-170 | 140-140 | 135-166 | 166-193 | 143-210 | 170-170 | 172-194 | 181-181 |
| Coratina 2 | 212-212 | 210-224 | 124-144 | 136-170 | 140-140 | 135-166 | 166-193 | 143-210 | 170-170 | 172-194 | 181-181 |
| Coratina 3 | 212-212 | 210-224 | 124-144 | 136-170 | 140-140 | 135-166 | 166-193 | 143-210 | 170-170 | 172-194 | 181-181 |
| Cornacciola | 212-222 | 214-214 | 126-144 | 150-159 | 150-150 | 143-143 | 166-177 | 156-205 | 205-205 | 176-182 | 181-185 |
| Corneglia | 218-222 | 214-224 | 126-126 | 136-170 | 140-140 | 143-143 | 166-177 | 154-182 | 213-220 | 172-198 | 181-181 |
| Cornia | 214-218 | 218-218 | 130-144 | 184-197 | 140-140 | 143-143 | 164-177 | 143-154 | 164-213 | 198-198 | 181-181 |
| Corniola | 208-212 | 212-212 | 124-144 | 170-184 | 144-144 | 143-143 | 177-193 | 143-205 | 200-200 | 172-182 | 177-179 |
| Corniolo | 214-222 | 214-214 | 130-144 | 150-157 | 140-140 | 135-143 | 177-193 | 161-205 | 205-205 | 182-206 | 179-181 |
| Cornula | 208-212 | 210-214 | 124-144 | 157-157 | 140-140 | 150-150 | 177-193 | 182-205 | 220-243 | 162-182 | 179-185 |
| Coroncina | 212-222 | 212-214 | 130-144 | 170-184 | 150-150 | 135-135 | 177-193 | 161-182 | 200-200 | 172-194 | 181-185 |
| Correggiolo | 208-212 | 214-224 | 124-144 | 159-170 | 144-144 | 143-143 | 177-182 | 182-205 | 205-205 | 172-194 | 179-181 |
| Correggiolo di Montegridolfo | 208-212 | 214-224 | 124-144 | 159-170 | 144-144 | 143-143 | 177-182 | 182-205 | 205-205 | 172-194 | 179-181 |
| Correggiolo di Pallese | 208-212 | 214-224 | 124-144 | 159-170 | 144-144 | 143-143 | 177-182 | 182-205 | 205-205 | 172-194 | 179-181 |
| Corsicana da mensa | 214-222 | 212-221 | 126-144 | 157-159 | 150-150 | 143-143 | 166-166 | 150-161 | 213-213 | 176-182 | 179-181 |
| Corsicana da olio | 212-222 | 214-224 | 126-144 | 159-184 | 144-150 | 143-143 | 177-182 | 182-210 | 185-185 | 182-198 | 181-185 |
| Craputea | 214-214 | 218-218 | 126-126 | 157-159 | 150-150 | 143-143 | 177-193 | 182-182 | 185-185 | 172-194 | 179-185 |
| Crastu | 208-208 | 214-214 | 124-144 | 157-170 | 144-144 | 150-150 | 177-177 | 182-182 | 185-185 | 182-198 | 179-181 |
| Crognolo | 214-222 | 218-224 | 124-144 | 157-170 | 150-150 | 150-150 | 177-182 | 182-210 | 164-164 | 176-198 | 179-181 |
| CSM | 208-212 | 214-224 | 124-124 | 159-159 | 144-144 | 150-150 | 177-177 | 182-210 | 213-213 | 162-206 | 177-177 |
| CSS-02-Minerva | 208-212 | 210-214 | 130-144 | 184-184 | 144-144 | 150-150 | 166-166 | 154-205 | 205-205 | 162-206 | 177-179 |
| Cucca 1 | 208-212 | 214-214 | 124-144 | 170-170 | 144-144 | 150-150 | 177-193 | 182-182 | 213-213 | 162-198 | 179-181 |
| Cucca 2 | 212-212 | 214-214 | 124-144 | 170-170 | 144-144 | 150-182 | 177-177 | 182-210 | 205-205 | 162-198 | 179-181 |
| Curatora | 218-222 | 218-218 | 126-126 | 170-184 | 144-150 | 135-135 | 166-166 | 143-143 | 185-185 | 198-206 | 179-185 |
| Demoniata | 212-212 | 210-214 | 124-130 | 159-159 | 140-140 | 150-150 | 177-193 | 182-182 | 108-185 | 176-206 | 181-185 |
| Dolce Agogia | 212-218 | 218-218 | 124-124 | 184-197 | 140-140 | 143-143 | 177-193 | 143-143 | 185-213 | 172-182 | 179-181 |
| Dolce di Andria | 208-218 | 214-214 | 124-144 | 150-157 | 144-144 | 143-143 | 177-182 | 143-205 | 108-170 | 172-194 | 177-177 |
| Dolce di Cassano | 208-208 | 214-224 | 130-130 | 150-157 | 144-144 | 143-143 | 177-177 | 182-205 | 108-170 | 162-198 | 181-181 |
| Dolce di Cerchiara | 212-222 | 212-212 | 130-144 | 136-170 | 150-150 | 143-143 | 166-193 | 143-205 | 205-205 | 162-198 | 179-181 |
| Dolce di Rossano | 212-212 | 214-214 | 130-144 | 150-159 | 144-144 | 143-143 | 177-177 | 154-182 | 205-205 | 172-182 | 177-179 |
| **Don Carlo** | **212-212** | **221-221** | **124-144** | **136-157** | **144-144** | **150-150** | **166-193** | **154-205** | **125-200** | **198-206** | **177-181** |
| Donna Francesca | 208-222 | 210-224 | 124-144 | 136-159 | 144-144 | 150-150 | 177-193 | 154-205 | 170-205 | 182-198 | 179-179 |
| Dritta | 208-212 | 214-214 | 124-130 | 159-170 | 140-140 | 143-143 | 166-193 | 182-182 | 220-220 | 172-176 | 169-179 |
| Dritta Loreto | 214-222 | 214-224 | 144-144 | 159-159 | 144-144 | 150-150 | 177-193 | 182-210 | 185-220 | 172-206 | 181-185 |
| Emilia | 208-212 | 214-214 | 124-144 | 159-170 | 144-144 | 150-150 | 156-193 | 182-182 | 213-213 | 172-206 | 177-179 |
| Erbano 1 | 212-212 | 210-212 | 124-144 | 159-159 | 140-140 | 150-150 | 177-193 | 182-182 | 108-108 | 172-182 | 173-177 |
| Erbano 2 | 212-222 | 214-250 | 124-144 | 159-170 | 140-140 | 150-150 | 177-193 | 143-182 | 213-213 | 172-182 | 173-177 |
| Faresana | 212-212 | 214-214 | 124-126 | 159-184 | 150-150 | 143-143 | 166-177 | 150-150 | 205-205 | 182-198 | 179-181 |
| Faresana | 208-208 | 214-214 | 124-126 | 159-184 | 140-140 | 143-143 | 166-177 | 143-161 | 205-205 | 182-198 | 179-181 |
| Fasolina | 214-222 | 214-214 | 126-144 | 150-159 | 150-150 | 150-150 | 177-177 | 154-205 | 205-205 | 198-206 | 179-179 |
| Fasolona | 214-214 | 214-214 | 130-130 | 159-184 | 150-150 | 150-150 | 177-193 | 154-154 | 205-205 | 198-206 | 179-181 |
| Favarol | 208-208 | 214-214 | 126-144 | 150-157 | 150-150 | 143-143 | 166-177 | 154-210 | 185-185 | 182-206 | 177-177 |
| Fecciaro | 214-222 | 214-221 | 124-130 | 159-159 | 140-140 | 143-143 | 166-177 | 143-182 | 205-205 | 176-198 | 179-181 |
| Feglina | 212-212 | 212-212 | 126-144 | 170-203 | 150-150 | 135-135 | 177-193 | 143-205 | 164-213 | 162-176 | 181-181 |
| Femminella di Torraca | 218-222 | 214-218 | 130-144 | 157-159 | 140-140 | 143-143 | 164-177 | 143-143 | 164-213 | 198-206 | 181-181 |
| Filare | 212-222 | 214-214 | 124-130 | 150-150 | 144-144 | 150-150 | 177-193 | 154-205 | 213-213 | 184-206 | 179-185 |
| Fosco | 208-212 | 214-224 | 124-144 | 159-170 | 144-144 | 150-150 | 177-177 | 182-210 | 205-205 | 172-194 | 179-181 |
| Frangivento | 208-208 | 214-214 | 124-130 | 159-170 | 144-144 | 135-166 | 177-177 | 154-205 | 205-232 | 162-176 | 179-185 |
| Frantoio 1 | 208-212 | 214-224 | 124-144 | 159-170 | 144-144 | 143-143 | 177-182 | 182-205 | 205-205 | 172-194 | 179-181 |
| Frantoio 10 | 208-212 | 214-224 | 124-144 | 159-170 | 144-144 | 143-143 | 177-182 | 182-205 | 205-205 | 172-194 | 179-181 |
| Frantoio 2 | 208-212 | 214-224 | 124-144 | 159-170 | 144-144 | 143-143 | 177-182 | 182-205 | 205-205 | 172-194 | 179-181 |
| Frantoio 3 | 208-212 | 214-224 | 124-144 | 159-170 | 144-144 | 143-143 | 177-182 | 182-205 | 205-205 | 172-194 | 179-181 |
| Frantoio 4 | 208-212 | 214-224 | 124-144 | 159-170 | 144-144 | 143-143 | 177-182 | 182-205 | 205-205 | 172-194 | 179-181 |
| Frantoio 5 | 208-212 | 214-224 | 124-144 | 159-170 | 144-144 | 143-143 | 177-182 | 182-205 | 205-205 | 172-194 | 179-181 |
| Frantoio 6 | 208-212 | 214-224 | 124-144 | 159-170 | 144-144 | 143-143 | 177-182 | 182-205 | 205-205 | 172-194 | 179-181 |
| Frantoio 7 | 208-212 | 214-224 | 124-144 | 159-170 | 144-144 | 143-143 | 177-182 | 182-205 | 205-205 | 172-194 | 179-181 |
| Frantoio 8 | 208-212 | 214-224 | 124-144 | 159-184 | 144-144 | 143-143 | 177-182 | 182-205 | 205-205 | 172-194 | 179-181 |
| Frantoio 9 | 208-212 | 214-224 | 124-144 | 159-184 | 144-144 | 143-143 | 177-182 | 182-205 | 205-205 | 172-194 | 179-181 |
| Frantoio di Montegridolfo | 208-212 | 214-224 | 124-144 | 159-170 | 144-144 | 143-143 | 177-182 | 182-205 | 205-205 | 172-194 | 179-181 |
| Frantoio di Villa Verrucchio | 208-212 | 214-224 | 124-144 | 159-170 | 144-144 | 143-143 | 177-182 | 182-205 | 205-205 | 172-194 | 179-181 |
| Frantoio FC | 208-212 | 214-224 | 124-144 | 159-170 | 144-144 | 143-143 | 177-182 | 182-205 | 205-205 | 172-194 | 179-181 |
| Fs17 | 214-214 | 214-224 | 124-124 | 184-197 | 140-140 | 143-143 | 166-177 | 150-205 | 185-220 | 206-206 | 179-181 |
| Gaggiolo | 214-222 | 214-224 | 126-144 | 159-170 | 150-150 | 143-143 | 177-182 | 182-205 | 185-185 | 182-206 | 179-179 |
| **Gentile dell'Aquila** | **212-212** | **214-214** | **144-144** | **150-184** | **140-140** | **182-182** | **166-193** | **182-205** | **213-213** | **198-210** | **175-181** |
| Gentile di Chieti | 208-212 | 210-214 | 124-130 | 170-170 | 140-140 | 143-143 | 166-193 | 154-205 | 213-213 | 172-172 | 169-179 |
| Gentile di Larino 1 | 208-212 | 214-214 | 124-130 | 150-184 | 140-140 | 150-150 | 166-193 | 182-182 | 213-213 | 172-194 | 169-179 |
| Gentile di Larino 5 | 208-212 | 214-214 | 124-130 | 150-184 | 140-140 | 150-150 | 166-193 | 182-182 | 213-213 | 172-194 | 169-179 |
| Gentile nera di Colletorto | 208-212 | 214-214 | 124-126 | 136-170 | 140-140 | 135-135 | 177-193 | 154-205 | 213-213 | 198-210 | 177-179 |
| Ghiacciolo | 214-214 | 214-224 | 126-144 | 157-184 | 140-140 | 135-135 | 166-166 | 150-161 | 185-232 | 198-206 | 177-179 |
| Ghiacciora | 212-212 | 214-214 | 124-144 | 136-157 | 150-150 | 135-135 | 166-177 | 150-210 | 205-205 | 162-176 | 179-181 |
| Ghiandaro | 208-212 | 214-214 | 124-144 | 159-170 | 144-144 | 150-150 | 177-193 | 154-205 | 205-205 | 166-182 | 163-177 |
| Ghiannara | 212-218 | 214-224 | 124-144 | 170-184 | 144-144 | 150-150 | 177-177 | 182-210 | 205-205 | 176-206 | 179-185 |
| Giardino | 214-222 | 218-218 | 126-144 | 170-184 | 150-150 | 150-150 | 166-193 | 182-182 | 213-213 | 182-206 | 181-181 |
| Giarfara | 212-212 | 214-214 | 144-144 | 157-184 | 140-140 | 150-150 | 166-193 | 182-182 | 108-108 | 176-182 | 179-179 |
| Giarraffa 1 | 212-212 | 210-214 | 124-144 | 136-159 | 144-144 | 150-150 | 166-166 | 143-182 | 213-213 | 162-198 | 181-185 |
| Giarraffa 2 | 212-212 | 214-214 | 144-144 | 150-184 | 140-140 | 150-150 | 166-193 | 182-182 | 108-108 | 172-182 | 177-181 |
| Ginestrino | 208-212 | 214-224 | 144-144 | 159-184 | 144-144 | 150-150 | 177-193 | 182-205 | 220-220 | 162-198 | 181-181 |
| Giusta | 214-222 | 214-214 | 126-144 | 170-184 | 144-144 | 143-143 | 177-177 | 182-182 | 205-205 | 162-198 | 177-179 |
| Gnagnaro | 212-214 | 210-214 | 124-144 | 170-170 | 144-144 | 150-182 | 166-166 | 154-182 | 220-243 | 162-206 | 175-175 |
| Grappolo | 208-218 | 210-218 | 124-144 | 170-184 | 150-150 | 150-150 | 156-182 | 182-182 | 220-220 | 182-206 | 181-181 |
| Grappuda | 212-212 | 218-218 | 130-144 | 184-203 | 140-140 | 150-150 | 166-193 | 161-205 | 213-232 | 172-182 | 177-181 |
| Gremignolo | 208-212 | 214-224 | 124-144 | 170-184 | 144-144 | 150-150 | 156-193 | 182-205 | 205-205 | 194-206 | 177-179 |
| Gremignolo di Bolgheri | 222-222 | 214-224 | 144-144 | 159-184 | 144-144 | 150-150 | 156-193 | 182-205 | 205-205 | 194-206 | 177-179 |
| Grignan | 212-212 | 212-221 | 124-130 | 157-157 | 140-140 | 150-150 | 166-166 | 182-210 | 213-220 | 194-198 | 177-185 |
| Grossa di Cassano | 208-218 | 210-214 | 124-144 | 136-159 | 140-140 | 143-143 | 166-177 | 182-182 | 205-232 | 162-182 | 179-179 |
| Grossa di Gerace | 212-212 | 212-212 | 126-144 | 150-170 | 140-150 | 143-143 | 166-177 | 143-182 | 200-200 | 182-198 | 179-181 |
| Grossa di Spagna | 208-208 | 210-214 | 124-144 | 136-184 | 144-144 | 143-143 | 166-166 | 143-245 | 108-108 | 162-172 | 177-177 |
| Grossa di Venafro | 212-222 | 214-214 | 124-130 | 150-150 | 144-144 | 150-150 | 177-193 | 154-205 | 213-213 | 162-204 | 177-177 |
| Grossale | 214-214 | 214-214 | 126-144 | 157-159 | 144-144 | 143-150 | 166-166 | 143-143 | 205-232 | 194-198 | 179-181 |
| I 77 | 218-222 | 218-218 | 124-130 | 150-157 | 140-140 | 135-135 | 166-177 | 154-205 | 205-205 | 198-206 | 179-179 |
| Iacona 1 | 208-212 | 214-214 | 124-144 | 159-184 | 144-144 | 150-150 | 166-193 | 182-182 | 108-205 | 162-162 | 179-181 |
| Iacona 2 | 208-212 | 214-214 | 124-144 | 159-184 | 144-144 | 150-150 | 166-193 | 182-182 | 108-205 | 162-162 | 179-181 |
| **Imperiale** | **208-212** | **210-250** | **130-144** | **159-184** | **144-144** | **150-150** | **166-193** | **143-143** | **213-232** | **182-198** | **177-185** |
| Intosso | 212-222 | 210-214 | 124-130 | 159-184 | 140-140 | 166-166 | 166-166 | 154-205 | 205-205 | 162-210 | 179-181 |
| Itrana | 214-218 | 214-224 | 130-144 | 159-170 | 150-150 | 143-143 | 166-177 | 150-161 | 220-220 | 182-198 | 179-181 |
| Larcianese | 208-218 | 212-221 | 124-144 | 159-170 | 150-150 | 143-143 | 166-177 | 161-205 | 200-200 | 182-206 | 181-181 |
| **Lastrino** | **212-218** | **210-210** | **124-130** | **184-203** | **144-144** | **135-135** | **156-166** | **156-161** | **220-232** | **194-198** | **179-179** |
| Laurina | 214-214 | 214-224 | 124-144 | 157-157 | 144-144 | 143-143 | 177-193 | 154-205 | 200-200 | 172-172 | 177-185 |
| **Lavagnina** | **212-212** | **210-214** | **130-144** | **136-170** | **144-144** | **150-182** | **166-193** | **143-245** | **213-232** | **172-176** | **179-181** |
| Lazzera Reale | 212-214 | 214-214 | 124-126 | 157-157 | 140-140 | 150-150 | 156-166 | 154-182 | 220-232 | 162-206 | 179-181 |
| Lea | 214-214 | 218-224 | 130-144 | 159-159 | 140-140 | 143-150 | 166-193 | 154-210 | 205-232 | 198-206 | 179-181 |
| Leccino 1 | 212-212 | 214-214 | 124-130 | 150-150 | 144-144 | 143-143 | 166-193 | 154-205 | 213-213 | 162-206 | 177-177 |
| Leccino 10 | 212-212 | 214-224 | 124-130 | 150-150 | 144-144 | 143-143 | 166-193 | 154-205 | 213-213 | 162-206 | 177-177 |
| Leccino 2 | 212-222 | 214-224 | 124-144 | 150-150 | 144-144 | 143-143 | 177-193 | 154-205 | 213-220 | 162-206 | 177-177 |
| Leccino 3 | 212-222 | 214-214 | 124-130 | 150-150 | 144-144 | 143-143 | 166-193 | 154-205 | 213-213 | 162-206 | 177-177 |
| Leccino 4 | 212-222 | 214-214 | 124-130 | 150-150 | 144-144 | 143-143 | 166-193 | 154-205 | 213-213 | 162-206 | 177-177 |
| Leccino 5 | 212-222 | 214-224 | 124-130 | 150-150 | 144-144 | 143-150 | 177-193 | 154-154 | 213-213 | 162-206 | 177-177 |
| Leccino 6 | 212-222 | 214-224 | 126-144 | 184-203 | 144-144 | 150-150 | 166-193 | 182-210 | 205-205 | 162-206 | 177-177 |
| Leccino 7 | 212-222 | 214-224 | 126-144 | 184-203 | 144-144 | 150-150 | 166-193 | 182-210 | 205-205 | 162-206 | 177-177 |
| Leccino 8 | 212-222 | 214-224 | 126-144 | 184-203 | 144-144 | 150-150 | 166-193 | 182-210 | 205-205 | 162-206 | 177-177 |
| Leccino 9 | 212-222 | 214-224 | 126-144 | 184-203 | 144-144 | 150-150 | 166-193 | 182-210 | 205-205 | 162-206 | 177-177 |
| Leccino Compatto | 212-222 | 214-224 | 124-144 | 150-150 | 144-144 | 143-143 | 177-193 | 154-205 | 213-213 | 162-206 | 177-177 |
| Leccino Dwarf | 208-212 | 214-224 | 124-144 | 170-184 | 144-144 | 150-150 | 166-193 | 182-210 | 205-205 | 162-206 | 177-177 |
| Leccino Minerva 1 | 208-212 | 214-224 | 124-144 | 170-184 | 144-144 | 150-150 | 166-193 | 182-210 | 205-205 | 162-206 | 177-177 |
| Leccino Minerva 2 | 212*-212 | 214-224 | 144-144 | 159-203 | 144-144 | 150-150 | 166-193 | 182-210 | 205-205 | 162-206 | 177-177 |
| Leccino Pisa 4 | 208-212 | 214-224 | 124-144 | 170-184 | 144-144 | 150-150 | 166-193 | 182-210 | 205-205 | 162-206 | 177-177 |
| Leccino Pisa 9 | 208-212 | 214-224 | 124-144 | 170-184 | 144-144 | 150-150 | 166-193 | 182-210 | 205-205 | 162-206 | 177-177 |
| Leccio del Corno 1 | 208-212 | 214-224 | 124-130 | 150-150 | 144-144 | 166-166 | 177-177 | 154-205 | 205-205 | 182-206 | 181-181 |
| Leccio del Corno 2 | 208-212 | 214-214 | 144-144 | 159-184 | 144-144 | 150-150 | 166-193 | 182-210 | 205-205 | 182-206 | 181-181 |
| Leccio Marammano 1 | 208-212 | 214-224 | 124-144 | 170-184 | 144-144 | 150-150 | 166-193 | 182-182 | 205-205 | 198-206 | 177-181 |
| Leccio Maremmano 2 | 208-212 | 214-224 | 124-144 | 170-184 | 144-144 | 150-150 | 166-193 | 182-210 | 205-205 | 198-206 | 177-181 |
| Leccione | 208-212 | 214-224 | 124-124 | 159-159 | 144-144 | 150-150 | 177-177 | 182-210 | 205-232 | 172-206 | 179-185 |
| Lezze | 208-218 | 210-210 | 124-144 | 136-159 | 140-140 | 135-135 | 177-182 | 143-161 | 164-185 | 172-194 | 181-181 |
| Lumiaru | 208-212 | 214-214 | 124-144 | 159-184 | 144-144 | 143-143 | 166-193 | 143-182 | 205-205 | 176-206 | 177-181 |
| Mafra | 212-212 | 212-212 | 126-144 | 136-159 | 144-144 | 143-143 | 166-182 | 143-143 | 213-213 | 162-198 | 179-179 |
| Mafra di Cerchiara | 208-222 | 212-221 | 126-144 | 136-159 | 144-144 | 143-143 | 166-177 | 143-205 | 213-213 | 162-182 | 179-185 |
| Maglianese | 212-212 | 214-224 | 126-144 | 157-157 | 140-140 | 143-143 | 166-182 | 154-210 | 185-232 | 198-206 | 177-181 |
| Maiatica di Ferrantina 1 | 208-208 | 214-214 | 124-144 | 170-184 | 144-144 | 143-143 | 166-193 | 182-182 | 205-205 | 198-206 | 177-179 |
| Maiatica di Ferrantina 2 | 208-208 | 214-214 | 124-144 | 170-184 | 144-144 | 143-143 | 166-193 | 182-182 | 205-205 | 198-206 | 177-179 |
| Majorca | 214-214 | 214-224 | 126-144 | 170-184 | 150-150 | 135-135 | 166-193 | 150-150 | 213-220 | 162-172 | 181-185 |
| Mandanici | 208-212 | 210-214 | 124-130 | 159-170 | 144-144 | 150-150 | 177-193 | 143-210 | 164-205 | 162-182 | 179-181 |
| Manna | 214-214 | 214-224 | 126-144 | 170-184 | 150-150 | 135-135 | 166-193 | 150-150 | 213-220 | 162-172 | 181-185 |
| Mantonica | 208-222 | 214-214 | 124-126 | 157-159 | 144-144 | 150-150 | 177-177 | 182-182 | 108-205 | 172-182 | 177-181 |
| Marchiaio | 208-208 | 210-210 | 124-144 | 150-157 | 150-150 | 166-166 | 164-182 | 182-182 | 125-125 | 194-194 | 179-181 |
| Marina | 212-222 | 214-214 | 126-126 | 184-197 | 144-144 | 135-135 | 166-193 | 154-154 | 213-232 | 162-198 | 179-181 |
| Marinese | 212-214 | 212-224 | 124-144 | 136-136 | 144-144 | 135-143 | 166-166 | 143-210 | 205-213 | 162-194 | 179-179 |
| Marzio | 208-218 | 210-218 | 130-130 | 159-159 | 150-150 | 150-150 | 166-182 | 182-205 | 200-220 | 162-198 | 181-181 |
| Maurino 1 | 208-208 | 214-224 | 144-144 | 159-184 | 144-144 | 150-150 | 177-182 | 182-210 | 220-220 | 162-198 | 181-181 |
| Maurino 2 | 208-212 | 214-224 | 144-144 | 184-184 | 144-144 | 150-150 | 177-193 | 182-210 | 220-220 | 162-198 | 181-181 |
| Maurino 3 | 208-212 | 214-224 | 144-144 | 159-184 | 144-144 | 150-150 | 177-182 | 182-210 | 220-220 | 162-198 | 181-181 |
| Maurino 4 | 208-212 | 214-224 | 130-144 | 159-184 | 144-144 | 150-150 | 177-193 | 182-210 | 220-220 | 162-198 | 181-181 |
| Mele | 208-208 | 214-214 | 124-144 | 150-157 | 144-144 | 143-143 | 166-177 | 150-182 | 108-170 | 162-182 | 177-185 |
| Mignola | 214-218 | 214-221 | 126-144 | 157-157 | 144-144 | 143-143 | 177-193 | 154-205 | 200-200 | 172-172 | 177-185 |
| Mignolo | 208-218 | 210-218 | 124-144 | 159-170 | 150-150 | 150-150 | 156-177 | 182-205 | 200-200 | 182-206 | 181-181 |
| Mignolo Cerretano | 208-218 | 214-224 | 124-126 | 150-159 | 140-140 | 143-143 | 177-177 | 182-205 | 164-164 | 162-172 | 175-181 |
| Minna di Vacca | 208-212 | 214-214 | 124-130 | 159-184 | 140-140 | 150-150 | 166-193 | 182-182 | 108-108 | 176-206 | 181-185 |
| Minuta 1 | 208-208 | 210-214 | 126-126 | 157-157 | 144-144 | 143-166 | 177-193 | 182-182 | 170-213 | 176-198 | 179-179 |
| Minuta 2 | 208-212 | 210-214 | 124-144 | 159-170 | 144-144 | 143-166 | 177-177 | 154-205 | 170-170 | 162-198 | 179-179 |
| Minuta3 | 212-212 | 214-221 | 124-124 | 159-159 | 150-150 | 150-150 | 177-193 | 154-245 | 164-213 | 176-206 | 181-181 |
| Minutella | 214-214 | 214-224 | 124-130 | 136-150 | 144-150 | 135-135 | 166-166 | 150-161 | 205-205 | 162-198 | 179-181 |
| Monaca | 208-212 | 210-214 | 124-144 | 157-159 | 144-144 | 150-150 | 177-193 | 154-182 | 164-205 | 176-206 | 181-181 |
| Mora | 208-212 | 210-224 | 124-126 | 170-184 | 144-144 | 143-143 | 166-193 | 143-210 | 108-164 | 162-182 | 179-181 |
| Moraiolo 1 | 212-222 | 214-214 | 124-130 | 150-150 | 144-144 | 182-182 | 177-193 | 154-205 | 213-213 | 184-206 | 179-185 |
| **Moraiolo 2** | **212-222** | **214-214** | **124-124** | **150-150** | **144-144** | **182-182** | **177-193** | **154-154** | **213-213** | **184-206** | **179-185** |
| Moraiolo 3 | 212-222 | 214-214 | 124-130 | 150-157 | 144-144 | 182-182 | 177-193 | 154-205 | 213-213 | 184-206 | 179-185 |
| Moraiolo 4 | 212-222 | 214-214 | 124-124 | 150-150 | 144-144 | 182-182 | 177-193 | 154-205 | 213-213 | 184-206 | 179-185 |
| Moraiolo 5 | 212-222 | 214-214 | 124-124 | 150-157 | 144-144 | 166-182 | 177-193 | 154-205 | 213-213 | 184-206 | 179-185 |
| Moraiolo T. Corsini | 212-222 | 214-214 | 124-130 | 150-157 | 144-144 | 166-182 | 177-193 | 154-205 | 213-220 | 184-206 | 179-185 |
| Morcaio | 212-218 | 210-210 | 126-144 | 150-150 | 150-150 | 143-143 | 156-193 | 154-182 | 205-205 | 182-206 | 185-185 |
| Morchiaio | 218-218 | 212-212 | 124-144 | 150-157 | 140-140 | 143-143 | 156-177 | 150-182 | 108-108 | 194-194 | 177-181 |
| Morcone | 208-222 | 210-214 | 124-126 | 157-157 | 144-144 | 135-166 | 177-193 | 182-205 | 220-243 | 176-176 | 177-181 |
| Morellona di Grecia | 212- 222 | 210-214 | 124-126 | 136-136 | 140-140 | 166-166 | 166-166 | 182-182 | 108-205 | 182-198 | 179-185 |
| Moresca 1 | 208-212 | 214-214 | 124-144 | 159-184 | 144-144 | 166-202 | 166-193 | 143-182 | 108-213 | 182-198 | 177-179 |
| Moresca 2 | 208-212 | 214-214 | 124-144 | 159-184 | 144-144 | 166-202 | 166-193 | 143-182 | 108-213 | 182-198 | 177-179 |
| Morinello | 212-214 | 218-218 | 130-144 | 150-157 | 140-140 | 150-150 | 177-193 | 154-182 | 200-200 | 182-206 | 179-181 |
| Murtiddara | 208-208 | 210-214 | 144-144 | 159-159 | 144-144 | 150-150 | 177-193 | 182-182 | 205-205 | 182-198 | 177-181 |
| Nasitana frutto grosso | 208-212 | 214-214 | 124-130 | 159-170 | 144-144 | 143-143 | 166-193 | 210-210 | 205-205 | 162-198 | 177-185 |
| Nebba | 208-208 | 214-214 | 144-144 | 150-184 | 140-140 | 150-150 | 166-193 | 182-182 | 205-205 | 176-206 | 181-185 |
| Nebbia | 208-212 | 210-214 | 124-130 | 159-184 | 140-140 | 135-135 | 177-177 | 154-210 | 205-205 | 172-206 | 177-181 |
| Nebbio di Chieti | 212- 222 | 214-214 | 124-130 | 159-159 | 144-144 | 135-135 | 177-193 | 154-205 | 213-213 | 172-210 | 175-179 |
| Nebbio di Pescara | 208-208 | 210-214 | 124-130 | 170-170 | 140-140 | 166-166 | 166-193 | 154-210 | 213-232 | 172-182 | 169-179 |
| Negrera | 214-214 | 214-224 | 126-144 | 170-184 | 150-150 | 143-143 | 166-193 | 182-182 | 200-200 | 162-198 | 179-179 |
| Nera di Cantinelle | 212-222 | 212-212 | 126-144 | 136-150 | 144-144 | 143-143 | 166-193 | 143-143 | 200-200 | 162-198 | 177-179 |
| Nera di Gonnos | 212-212 | 214-221 | 126-144 | 170-203 | 150-150 | 135-135 | 166-193 | 143-143 | 213-220 | 162-172 | 185-185 |
| Nera di Oliena | 214-222 | 212-218 | 126-144 | 157-159 | 144-150 | 143-143 | 164-177 | 150-161 | 213-213 | 176-182 | 179-181 |
| Nera di Villacidro | 214-222 | 214-221 | 126-144 | 157-159 | 144-150 | 143-143 | 164-177 | 150-161 | 213-213 | 182-198 | 179-181 |
| Nerba | 208-212 | 214-214 | 124-144 | 159-184 | 144-144 | 150-150 | 166-193 | 154-205 | 213-213 | 176-206 | 181-185 |
| Nerba Catanese | 208-212 | 214-214 | 124-144 | 159-184 | 144-144 | 150-150 | 166-193 | 154-182 | 213-213 | 162-172 | 177-185 |
| Noccioluta | 208-212 | 214-214 | 124-126 | 136-170 | 140-140 | 182-182 | 177-193 | 154-205 | 213-213 | 198-210 | 177-181 |
| Nocellara del Belice 1 | 212-212 | 214-214 | 144-144 | 150-184 | 140-140 | 166-166 | 166-193 | 182-182 | 108-205 | 162-172 | 177-181 |
| Nocellara del Belice 2 | 212-212 | 214-214 | 144-144 | 150-184 | 140-140 | 166-166 | 166-193 | 182-182 | 108-205 | 162-172 | 177-181 |
| Nocellara etnea | 212-212 | 210-210 | 124-144 | 159-184 | 144-144 | 150-150 | 166-193 | 143-182 | 220-220 | 172-182 | 181-181 |
| Nocellara etnea ovale | 212-212 | 210-210 | 124-144 | 159-184 | 144-144 | 143-143 | 166-193 | 143-182 | 220-220 | 198-206 | 181-185 |
| Nocellara messinese | 208-212 | 214-214 | 124-144 | 159-184 | 144-144 | 150-150 | 166-193 | 143-143 | 213-232 | 162-176 | 179-179 |
| Nocellara Nissena 1 | 208-212 | 210-214 | 124-144 | 136-159 | 144-144 | 150-150 | 166-193 | 154-154 | 205-205 | 176-198 | 179-179 |
| Nocellara Nissena 2 | 208-212 | 210-214 | 124-144 | 136-159 | 144-144 | 150-150 | 166-193 | 154-154 | 205-205 | 176-198 | 179-179 |
| Nociara | 212-222 | 210-214 | 124-126 | 170-170 | 140-140 | 143-143 | 166-193 | 182-182 | 164-232 | 162-162 | 179-185 |
| Nolca | 208-208 | 214-214 | 124-144 | 150-157 | 144-144 | 143-143 | 166-177 | 150-182 | 108-170 | 162-182 | 177-185 |
| Nostrale di Fiano | 212-222 | 214-224 | 126-144 | 159-170 | 144-144 | 143-143 | 177-182 | 182-210 | 185-185 | 182-206 | 179-181 |
| Nostrale di Rigali | 214-214 | 218-224 | 124-130 | 184-197 | 144-144 | 135-143 | 166-182 | 150-161 | 205-220 | 162-172 | 179-181 |
| Nostrana | 212-222 | 212-212 | 124-130 | 170-184 | 140-150 | 143-150 | 177-177 | 143-143 | 205-205 | 172-206 | 177-179 |
| Nostrana Campana | 218-222 | 218-218 | 126-144 | 184-197 | 140-140 | 135-150 | 166-182 | 143-210 | 164-213 | 194-198 | 181-181 |
| Nostrana di Brisighella 1 | 214-214 | 214-224 | 126-144 | 136-184 | 144-144 | 135-135 | 166-166 | 150-161 | 213-213 | 198-198 | 177-179 |
| Nostrana di Brisighella 2 | 212-212 | 214-214 | 124-130 | 136-157 | 144-144 | 135-135 | 166-193 | 182-210 | 205-220 | 198-198 | 177-179 |
| Nostrana di Brisighella 3 | 214-214 | 214-224 | 126-144 | 136-184 | 144-144 | 135-135 | 166-166 | 150-161 | 213-213 | 198-206 | 179-185 |
| Nostrana di Brisighella 4 | 212-212 | 214-214 | 124-126 | 136-157 | 144-144 | 135-135 | 166-193 | 182-210 | 205-220 | 198-198 | 177-179 |
| Ogliara | 214-222 | 214-214 | 124-126 | 150-157 | 140-140 | 143-143 | 164-193 | 161-161 | 213-232 | 172-198 | 179-185 |
| Ogliarola Barese | 208-212 | 214-224 | 124-144 | 159-170 | 144-144 | 143-143 | 177-182 | 182-205 | 205-205 | 172-194 | 179-181 |
| Ogliarola del Bradano 1 | 212-212 | 210-214 | 124-144 | 159-159 | 144-144 | 150-150 | 177-193 | 154-205 | 205-205 | 172-172 | 177-185 |
| Ogliarola del Bradano 2 | 208-212 | 214-224 | 124-144 | 159-170 | 144-144 | 143-143 | 177-182 | 182-205 | 205-205 | 172-194 | 179-181 |
| Ogliarola del Murge | 208-212 | 214-224 | 124-144 | 159-184 | 144-144 | 150-150 | 177-177 | 182-205 | 205-205 | 172-198 | 181-181 |
| Ogliarola di Montalbano | 208-212 | 214-224 | 124-144 | 159-170 | 144-144 | 143-143 | 177-182 | 182-205 | 205-205 | 172-194 | 179-181 |
| Ogliarola Garganica | 208-212 | 214-224 | 124-144 | 159-170 | 144-144 | 143-143 | 177-182 | 182-205 | 205-205 | 172-194 | 179-181 |
| Ogliarola Messinese 1 | 208-212 | 214-214 | 124-144 | 159-184 | 144-144 | 166-182 | 166-193 | 182-182 | 108-205 | 162-162 | 169-181 |
| Ogliarola Messinese 2 | 208-212 | 214-214 | 124-144 | 159-184 | 144-144 | 166-182 | 166-193 | 182-182 | 108-205 | 182-198 | 177-177 |
| Ogliarola Salentina | 212-212 | 210-214 | 124-144 | 159-159 | 144-144 | 143-143 | 177-193 | 150-182 | 170-170 | 162-162 | 173-177 |
| OgliarolaVulture | 212-218 | 214-214 | 124-124 | 150-184 | 144-150 | 143-143 | 166-193 | 182-182 | 213-213 | 172-182 | 179-181 |
| Ogliastro grande | 214-218 | 218-218 | 126-126 | 159-159 | 144-144 | 150-150 | 164-164 | 143-143 | 200-220 | 182-206 | 181-185 |
| Olianedda | 214-222 | 214-221 | 126-144 | 157-159 | 144-144 | 143-143 | 166-177 | 150-161 | 213-213 | 176-182 | 179-181 |
| Oliastro | 208-218 | 210-210 | 126-144 | 136-159 | 140-140 | 143-143 | 166-177 | 143-161 | 164-185 | 172-194 | 181-181 |
| Olieddu | 214-222 | 214-218 | 130-144 | 170-184 | 144-150 | 143-143 | 177-193 | 161-205 | 205-205 | 172-194 | 181-185 |
| Oliva Bianca | 218-222 | 218-224 | 124-130 | 150-157 | 144-144 | 135-135 | 166-193 | 205-205 | 200-220 | 172-206 | 177-181 |
| Oliva da polpa | 214-222 | 218-218 | 124-126 | 150-157 | 140-150 | 150-150 | 164-182 | 161-161 | 213-213 | 172-198 | 177-185 |
| Oliva grossa | 214-214 | 214-224 | 126-144 | 136-184 | 140-140 | 150-150 | 166-166 | 150-161 | 205-205 | 198-206 | 179-179 |
| Oliva Rossa | 208-218 | 210-210 | 124-144 | 136-159 | 140-140 | 135-150 | 177-177 | 143-182 | 164-185 | 172-194 | 181-181 |
| Oliva torsa | 212-214 | 218-224 | 124-130 | 136-136 | 144-144 | 135-135 | 164-164 | 143-210 | 205-220 | 172-194 | 181-181 |
| Oliva vera | 214-214 | 218-218 | 130-144 | 150-157 | 144-144 | 135-135 | 177-177 | 154-154 | 213-213 | 198-206 | 179-185 |
| Olivago | 212-212 | 212-212 | 126-144 | 150-157 | 144-144 | 135-135 | 166-166 | 161-210 | 185-185 | 172-198 | 177-181 |
| Olivastra di Populonia | 212-222 | 212-218 | 124-130 | 150-150 | 150-150 | 182-182 | 156-164 | 156-161 | 220-232 | 182-206 | 181-181 |
| Olivastra Seggianese | 212-218 | 210-212 | 126-144 | 150-159 | 150-150 | 150-150 | 156-164 | 156-182 | 205-205 | 182-206 | 179-185 |
| **Olivastro d'Aprile** | **222-222** | **210-214** | **124-126** | **159-159** | **144-144** | **135-182** | **166-166** | **205-205** | **213-213** | **162-172** | **173-175** |
| Olivastro di Bucchianico | 208-222 | 210-214 | 124-124 | 159-170 | 140-140 | 182-182 | 177-193 | 154-210 | 213-213 | 198-210 | 179-181 |
| **Olivastro di Montenero** | **218-222** | **210-214** | **121-121** | **170-170** | **144-144** | **135-166** | **177-193** | **210-210** | **170-213** | **162-182** | **173-177** |
| Olivastro Dritto | 212-212 | 210-214 | 124-144 | 159-159 | 144-144 | 150-150 | 177-193 | 154-210 | 205-205 | 166-172 | 163-177 |
| Olivastro Frentano | 208-222 | 210-214 | 126-144 | 170-170 | 140-140 | 182-182 | 177-177 | 154-210 | 205-232 | 182-206 | 177-181 |
| Olivella appuntita | 214-222 | 212-214 | 126-144 | 170-184 | 140-140 | 143-143 | 164-177 | 154-161 | 205-205 | 162-194 | 179-185 |
| Olivella di Cerchiara | 212-218 | 212-212 | 124-144 | 136-159 | 144-144 | 143-143 | 166-177 | 143-182 | 213-213 | 198-206 | 177-179 |
| Olivella di Grottaminarda | 214-218 | 214-214 | 124-126 | 150-157 | 150-150 | 135-166 | 166-177 | 150-161 | 200-200 | 182-206 | 177-181 |
| Olivetta Nera | 212- 222 | 214-214 | 124-130 | 150-150 | 144-144 | 150-150 | 177-193 | 154-205 | 205-205 | 162-188 | 173-183 |
| Olivo da olio | 214-214 | 218-218 | 124-130 | 150-157 | 140-144 | 143-150 | 164-177 | 182-182 | 213-213 | 172-198 | 179-185 |
| Olivo da salare | 214-222 | 214-214 | 126-130 | 150-157 | 140-140 | 135-135 | 166-177 | 154-154 | 200-200 | 162-194 | 179-181 |
| Olivo del mulino | 212-214 | 214-224 | 130-144 | 159-159 | 150-150 | 182-182 | 156-193 | 150-161 | 213-213 | 176-176 | 177-181 |
| Olivo della Madonna | 212-222 | 214-214 | 124-144 | 170-170 | 140-140 | 150-150 | 177-177 | 182-210 | 205-205 | 162-206 | 177-177 |
| Olivo delle Alpi | 214-222 | 214-224 | 126-144 | 184-203 | 150-150 | 150-150 | 166-177 | 161-161 | 200-200 | 162-198 | 179-185 |
| Olivo di Casavecchia | 212-212 | 212-221 | 124-124 | 150-157 | 150-150 | 143-143 | 164-166 | 161-205 | 200-200 | 182-194 | 179-181 |
| Olivo di Castiglione | 208-212 | 214-214 | 124-144 | 159-184 | 144-144 | 150-150 | 166-193 | 154-182 | 213-213 | 198-206 | 181-185 |
| Olivo di San Lorenzo | 208-218 | 214-221 | 144-144 | 150-150 | 150-150 | 150-150 | 166-193 | 161-205 | 200-220 | 162-198 | 181-181 |
| Olivoce | 212-218 | 214-214 | 124-126 | 136-159 | 140-140 | 182-182 | 166-193 | 154-205 | 213-232 | 198-210 | 177-181 |
| Olivomensa | 212-212 | 214-214 | 124-126 | 159-184 | 144-144 | 143-143 | 166-177 | 150-150 | 209-209 | 182-198 | 179-181 |
| Olivona | 212-212 | 214-214 | 124-126 | 157-157 | 140-140 | 150-150 | 166-166 | 143-143 | 213-213 | 182-198 | 179-179 |
| Olivone di Viterbo | 214-222 | 218-224 | 124-130 | 157-157 | 150-150 | 135-143 | 166-177 | 143-182 | 185-185 | 172-182 | 177-179 |
| OperaPia | 212-222 | 214-221 | 124-144 | 136-184 | 150-150 | 150-150 | 166-193 | 154-154 | 209-209 | 162-172 | 179-181 |
| Orazio | 218-222 | 214-214 | 126-144 | 159-170 | 144-144 | 143-143 | 177-177 | 150-205 | 209-209 | 198-198 | 179-181 |
| Orbetana | 214-218 | 218-224 | 124-144 | 184-197 | 140-140 | 135-135 | 166-177 | 182-210 | 200-200 | 162-182 | 177-179 |
| Ornellaia | 208-218 | 210-218 | 126-144 | 136-159 | 144-144 | 150-150 | 164-182 | 182-182 | 205-205 | 198-198 | 185-185 |
| Ortice | 218-218 | 214-224 | 126-144 | 150-157 | 140-140 | 135-166 | 166-182 | 161-205 | 164-164 | 194-198 | 177-185 |
| Ortolana | 214-218 | 218-218 | 121-124 | 170-184 | 140-140 | 143-143 | 164-164 | 161-161 | 205-213 | 162-194 | 177-181 |
| Ottobratica | 212-222 | 212-212 | 124-124 | 157-170 | 140-150 | 143-143 | 177-193 | 143-154 | 170-205 | 182-206 | 177-185 |
| Ottobrina | 214-214 | 218-224 | 126-144 | 159-170 | 150-150 | 150-150 | 166-193 | 150-205 | 200-220 | 182-194 | 181-181 |
| Paesana Bianca | 208-212 | 214-224 | 124-144 | 159-184 | 144-144 | 150-150 | 177-177 | 182-210 | 205-205 | 162-166 | 163-177 |
| Paesana Nera | 212-222 | 214-214 | 124-130 | 150-150 | 144-144 | 150-182 | 177-193 | 154-205 | 213-213 | 162-204 | 177-177 |
| Palma | 214-222 | 218-221 | 130-144 | 170-184 | 144-150 | 135-135 | 177-193 | 161-205 | 205-205 | 172-172 | 181-185 |
| Palmarola | 208-222 | 214-224 | 124-144 | 150-157 | 144-144 | 143-143 | 166-166 | 182-210 | 200-200 | 194-198 | 177-179 |
| Pampagliosa | 214-214 | 218-224 | 124-130 | 170-184 | 140-140 | 143-143 | 164-182 | 143-143 | 200-200 | 182-206 | 181-181 |
| Paschixedda | 214-222 | 212-221 | 126-144 | 157-159 | 144-150 | 143-143 | 164-177 | 150-161 | 213-213 | 176-182 | 179-181 |
| Pasola | 208-218 | 214-214 | 124-144 | 150-157 | 144-144 | 143-143 | 177-177 | 150-182 | 170-170 | 198-206 | 177-177 |
| Passulunara | 212-222 | 210-210 | 124-144 | 159-184 | 144-144 | 150-150 | 166-193 | 182-182 | 108-205 | 176-182 | 179-179 |
| Patrinostraru | 212-212 | 210-214 | 130-144 | 157-170 | 144-144 | 150-150 | 166-193 | 143-182 | 205-232 | 182-198 | 173-179 |
| Pendolino 1 | 212-222 | 214-214 | 126-130 | 150-159 | 144-144 | 166-166 | 177-177 | 154-205 | 205-205 | 162-206 | 181-181 |
| Pendolino 2 | 212-222 | 214-214 | 126-130 | 150-150 | 144-144 | 150-166 | 177-177 | 154-205 | 205-213 | 162-206 | 181-181 |
| Pendolino 3 | 222-222 | 214-214 | 126-130 | 150-150 | 144-144 | 166-166 | 177-182 | 154-205 | 205-205 | 162-206 | 181-181 |
| Pendolino 4 | 212-222 | 214-214 | 126-130 | 150-150 | 144-144 | 150-166 | 177-182 | 154-205 | 205-205 | 162-206 | 181-181 |
| Pendolino 5 | 212-222 | 214-214 | 126-130 | 150-150 | 144-144 | 166-166 | 177-177 | 154-205 | 205-213 | 162-206 | 181-181 |
| Pennulara | 212-222 | 214-214 | 130-144 | 150-159 | 140-150 | 143-143 | 177-193 | 143-182 | 209-220 | 198-206 | 179-179 |
| Peppino Leo | 208-218 | 214-214 | 124-144 | 150-159 | 144-144 | 135-135 | 177-182 | 143-205 | 108-108 | 172-194 | 177-177 |
| Peranzana | 212-222 | 212-214 | 130-144 | 170-170 | 144-144 | 182-182 | 166-193 | 143-205 | 108-205 | 162-172 | 181-181 |
| Perciasacchi | 212-214 | 214-224 | 124-144 | 136-136 | 150-150 | 143-143 | 164-177 | 161-182 | 213-220 | 162-198 | 181-181 |
| Pescarese | 208-212 | 214-224 | 124-126 | 159-170 | 144-144 | 143-143 | 177-193 | 154-205 | 220-243 | 198-206 | 179-185 |
| Pesciatino | 208-208 | 212-214 | 126-144 | 170-184 | 144-144 | 143-182 | 164-166 | 150-182 | 220-232 | 162-194 | 179-185 |
| **Pezz'e quaddu** | **214-214** | **214-221** | **126-144** | **136-203** | **144-150** | **135-135** | **166-166** | **150-205** | **213-213** | **162-162** | **181-185** |
| Piangente 1 | 208-212 | 214-214 | 126-126 | 157-184 | 144-144 | 150-150 | 166-193 | 154-154 | 205-205 | 162-206 | 181-181 |
| Piangente 2 | 212-222 | 214-214 | 124-130 | 150-150 | 144-144 | 150-150 | 177-193 | 154-205 | 205-205 | 162-206 | 181-181 |
| Piangente 3 | 208-212 | 214-224 | 144-144 | 159-184 | 144-144 | 150-150 | 166-193 | 182-210 | 205-205 | 162-206 | 181-181 |
| Piantone di Falerone | 218-222 | 218-218 | 124-130 | 150-157 | 144-144 | 135-135 | 166-177 | 210-210 | 205-232 | 182-182 | 177-185 |
| Piantone di Macerata | 212-222 | 218-224 | 124-144 | 170-184 | 144-144 | 143-143 | 166-193 | 182-210 | 185-200 | 162-198 | 179-181 |
| Piantone di Mogliano | 214-214 | 218-224 | 124-130 | 159-159 | 144-144 | 135-135 | 166-166 | 182-210 | 200-232 | 198-206 | 181-181 |
| Pidicuddara | 212-212 | 210-210 | 124-144 | 150-184 | 144-144 | 150-150 | 166-166 | 182-182 | 205-205 | 162-182 | 177-179 |
| Pignola | 214-222 | 214-214 | 126-130 | 150-157 | 150-150 | 143-143 | 177-193 | 154-182 | 205-205 | 182-206 | 181-185 |
| Pirunara 1 | 212-222 | 214-214 | 124-126 | 150-170 | 144-144 | 150-150 | 177-177 | 182-182 | 205-205 | 162-182 | 179-181 |
| Pirunara 2 | 212-222 | 214-214 | 124-126 | 150-170 | 144-144 | 150-150 | 177-177 | 182-182 | 205-205 | 162-182 | 179-181 |
| Pisciottana | 214-222 | 218-218 | 121-124 | 136-150 | 140-140 | 143-143 | 177-182 | 150-161 | 213-232 | 172-182 | 177-181 |
| Pizz'e Carroga | 212-222 | 218-218 | 126-144 | 170-184 | 140-140 | 135-135 | 164-193 | 150-150 | 125-125 | 162-172 | 179-185 |
| Pizzo di corvo 1 | 212-212 | 210-214 | 124-144 | 136-159 | 144-144 | 150-150 | 166-166 | 143-182 | 213-213 | 162-198 | 169-185 |
| Pizzo di corvo 2 | 212-212 | 210-214 | 124-144 | 136-159 | 140-140 | 150-150 | 166-166 | 143-182 | 213-213 | 172-182 | 175-179 |
| Pizzutella | 208-222 | 210-214 | 124-124 | 157-170 | 144-144 | 150-150 | 166-166 | 143-154 | 164-164 | 176-182 | 179-181 |
| Pocciolo | 218-222 | 218-218 | 124-130 | 150-150 | 140-140 | 135-135 | 166-182 | 154-205 | 205-205 | 176-206 | 181-185 |
| Posola | 212-218 | 214-214 | 124-144 | 170-184 | 144-144 | 135-166 | 166-193 | 154-205 | 213-213 | 182-206 | 173-181 |
| Posolella | 214-218 | 214-214 | 126-144 | 159-159 | 144-144 | 150-150 | 177-193 | 154-205 | 213-213 | 194-206 | 173-185 |
| Precoce | 208-212 | 214-214 | 126-126 | 159-170 | 140-140 | 150-150 | 166-193 | 182-182 | 205-232 | 172-198 | 169-179 |
| Procanica | 214-222 | 218-224 | 126-144 | 157-170 | 144-144 | 143-143 | 177-182 | 182-210 | 185-185 | 182-206 | 179-181 |
| Provenzale | 218-218 | 218-218 | 126-144 | 157-159 | 144-150 | 143-150 | 164-164 | 143-210 | 200-220 | 194-206 | 179-181 |
| Puntella | 222-222 | 214-214 | 124-130 | 159-159 | 144-144 | 202-202 | 166-193 | 154-205 | 213-213 | 194-198 | 177-185 |
| Punteruolo | 212-214 | 210-224 | 124-144 | 136-159 | 144-144 | 135-166 | 177-193 | 143-182 | 220-220 | 162-206 | 177-181 |
| Puntino | 208-212 | 214-224 | 124-144 | 159-184 | 144-144 | 150-150 | 177-177 | 182-205 | 205-205 | 172-206 | 175-181 |
| Racemo 1 | 212-212 | 210-224 | 124-144 | 136-159 | 140-140 | 135-166 | 166-193 | 143-210 | 170-170 | 172-194 | 181-181 |
| Racemo 2 | 208-208 | 210-224 | 124-144 | 136-159 | 140-140 | 135-166 | 166-193 | 143-210 | 170-170 | 172-194 | 181-181 |
| Racioppa | 208-208 | 212-214 | 124-130 | 159-170 | 144-144 | 143-143 | 166-193 | 161-205 | 220-232 | 172-182 | 179-185 |
| Racioppa | 212-222 | 214-214 | 124-124 | 157-157 | 144-144 | 143-150 | 166-177 | 150-154 | 205-220 | 182-206 | 181-185 |
| Racioppella | 218-222 | 212-224 | 124-130 | 170-184 | 144-144 | 135-135 | 164-182 | 143-143 | 205-220 | 194-198 | 177-179 |
| **Raggiola** | **212-222** | **218-224** | **126-144** | **157-170** | **144-144** | **150-150** | **177-182** | **161-182** | **185-185** | **182-206** | **179-181** |
| Raja | 214-222 | 218-218 | 124-130 | 150-157 | 140-140 | 135-143 | 177-193 | 161-161 | 213-232 | 182-206 | 179-181 |
| Raja Sabina | 212-222 | 218-224 | 124-144 | 157-159 | 144-144 | 150-150 | 177-182 | 182-210 | 185-185 | 176-198 | 179-181 |
| Rajo | 214-218 | 218-218 | 124-144 | 157-159 | 140-140 | 135-135 | 166-193 | 182-182 | 205-205 | 198-206 | 179-181 |
| Rastellina | 214-222 | 214-214 | 130-130 | 159-170 | 140-140 | 143-143 | 166-182 | 210-210 | 205-205 | 176-182 | 179-181 |
| **Ravece** | **214-218** | **212-224** | **124-144** | **136-136** | **144-150** | **135-135** | **164-177** | **143-150** | **205-205** | **162-194** | **181-185** |
| Raza | 212-222 | 214-224 | 126-144 | 159-170 | 150-150 | 143-143 | 177-182 | 182-210 | 185-185 | 182-206 | 179-179 |
| Razzaio | 212-218 | 210-214 | 124-130 | 157-157 | 140-140 | 150-150 | 166-193 | 154-205 | 205-232 | 182-206 | 181-181 |
| Razzo 1 | 208-212 | 214-224 | 124-144 | 159-170 | 144-144 | 150-150 | 177-177 | 154-205 | 205-205 | 182-206 | 181-185 |
| Razzo 2 | 208-212 | 210-214 | 124-130 | 150-170 | 140-144 | 150-150 | 177-177 | 154-205 | 205-205 | 182-206 | 181-185 |
| Razzola | 214-222 | 218-224 | 126-144 | 159-170 | 150-150 | 150-150 | 177-182 | 182-205 | 200-200 | 176-206 | 181-181 |
| Reale | 212-222 | 218-224 | 126-144 | 157-170 | 144-150 | 143-143 | 166-177 | 182-210 | 185-185 | 182-206 | 179-181 |
| Regina | 208-212 | 214-224 | 124-130 | 150-184 | 140-140 | 150-150 | 177-193 | 182-205 | 205-205 | 162-198 | 181-185 |
| Resciola di Venafro | 208-222 | 214-214 | 126-144 | 136-170 | 140-140 | 143-143 | 177-193 | 143-154 | 213-213 | 176-206 | 177-181 |
| Riminino | 214-222 | 214-224 | 126-144 | 157-157 | 144-144 | 143-182 | 166-177 | 143-182 | 205-205 | 176-198 | 177-181 |
| **Ritonnella** | **214-218** | **212-218** | **126-144** | **150-157** | **140-150** | **143-143** | **193-193** | **154-161** | **213-220** | **198-206** | **177-181** |
| Rizza | 212-222 | 214-214 | 126-144 | 159-159 | 144-144 | 150-150 | 166-182 | 143-210 | 125-200 | 182-198 | 179-181 |
| Rizzitella | 214-218 | 212-214 | 124-144 | 150-157 | 140-140 | 143-143 | 177-177 | 161-161 | 213-232 | 172-194 | 177-179 |
| Roma | 218-222 | 214-214 | 126-144 | 159-184 | 144-144 | 143-143 | 177-193 | 150-205 | 209-209 | 198-198 | 179-181 |
| Romanella | 208-222 | 214-218 | 124-144 | 159-170 | 144-144 | 143-143 | 177-182 | 182-205 | 200-200 | 172-172 | 179-185 |
| Romanella | 212-212 | 212-218 | 124-130 | 136-170 | 150-150 | 143-143 | 166-193 | 143-161 | 209-220 | 182-198 | 179-179 |
| Romanella Molisana | 212- 222 | 214-214 | 130-126 | 150-150 | 140-140 | 143-143 | 177-193 | 154-205 | 213-243 | 198-210 | 181-181 |
| Rosciola | 214-222 | 218-218 | 126-144 | 184-197 | 144-150 | 143-143 | 177-182 | 154-205 | 205-205 | 198-206 | 177-179 |
| Rosciola Coltodino | 208-218 | 212-212 | 126-144 | 184-184 | 150-150 | 182-202 | 177-182 | 156-161 | 200-200 | 182-206 | 179-181 |
| **Rosciola di Rotello** | **208-212** | **214-224** | **124-144** | **159-184** | **144-144** | **150-150** | **177-177** | **182-182** | **205-205** | **162-166** | **163-177** |
| Rosciola di Venafro | 222-222 | 214-214 | 124-124 | 150-184 | 144-144 | 202-202 | 177-193 | 154-205 | 243-243 | 198-206 | 177-181 |
| Rosciola laziale | 212-222 | 218-224 | 144-144 | 157-157 | 144-150 | 143-143 | 177-193 | 182-210 | 185-220 | 162-198 | 179-181 |
| Rosino | 222-222 | 212-214 | 124-130 | 184-184 | 140-140 | 150-150 | 156-193 | 154-205 | 220-243 | 182-206 | 185-185 |
| Rossellino | 208-208 | 210-212 | 126-144 | 170-184 | 144-144 | 202-202 | 164-166 | 156-205 | 220-232 | 172-194 | 179-185 |
| Rossello | 208-212 | 214-214 | 124-130 | 184-184 | 144-144 | 150-150 | 177-177 | 154-182 | 213-213 | 176-194 | 177-185 |
| Rossina | 214-222 | 214-214 | 126-144 | 150-157 | 150-150 | 135-135 | 177-193 | 161-210 | 213-232 | 176-182 | 177-181 |
| Rotondella | 212-222 | 214-214 | 130-144 | 150-150 | 140-140 | 150-150 | 166-166 | 154-182 | 200-200 | 182-198 | 179-181 |
| Rotondella di Foggia | 208-218 | 214-214 | 124-126 | 150-157 | 140-140 | 135-135 | 177-177 | 154-182 | 185-205 | 182-198 | 177-179 |
| Rotondella di Sanza 1 | 212-218 | 218-218 | 126-144 | 157-159 | 140-140 | 143-150 | 164-182 | 150-205 | 205-232 | 198-206 | 179-181 |
| Rotondella di Sanza 2 | 212-218 | 218-218 | 126-144 | 157-159 | 140-140 | 143-150 | 164-182 | 150-205 | 205-232 | 198-206 | 179-181 |
| **Rumignana** | **208-212** | **214-214** | **121-121** | **150-170** | **140-140** | **150-150** | **166-193** | **182-182** | **220-220** | **172-206** | **169-179** |
| **Rustica** | **212-222** | **210-214** | **124-126** | **150-184** | **140-140** | **135-135** | **166-166** | **154-205** | **205-205** | **198-206** | **173-173** |
| Ruveia | 214-214 | 214-218 | 124-126 | 170-184 | 140-140 | 143-143 | 164-182 | 154-205 | 213-232 | 172-194 | 181-185 |
| Salegna di Larino | 212-222 | 214-214 | 124-130 | 150-150 | 140-140 | 150-150 | 177-193 | 154-205 | 220-243 | 198-210 | 173-181 |
| Salella | 214-218 | 214-214 | 124-130 | 136-150 | 140-140 | 143-143 | 164-177 | 150-182 | 213-232 | 172-182 | 177-181 |
| Salicino | 212-222 | 210-214 | 124-126 | 136-159 | 144-144 | 150-150 | 156-193 | 182-205 | 205-205 | 162-198 | 179-181 |
| Salvia | 218-222 | 218-224 | 126-144 | 150-159 | 144-150 | 143-143 | 177-193 | 154-210 | 185-185 | 172-182 | 179-181 |
| Salviana | 214-222 | 214-224 | 130-144 | 150-157 | 140-140 | 135-135 | 177-182 | 182-210 | 185-220 | 182-206 | 181-185 |
| Sammartinara | 212-212 | 214-214 | 126-144 | 159-159 | 144-144 | 150-150 | 177-177 | 182-182 | 205-205 | 162-162 | 179-181 |
| Sammartinenga | 208-222 | 214-214 | 124-144 | 136-159 | 144-144 | 143-143 | 166-177 | 143-182 | 213-213 | 162-176 | 179-181 |
| San Benedetto | 208-212 | 214-214 | 124-144 | 157-157 | 144-144 | 135-143 | 166-193 | 154-182 | 205-205 | 162-198 | 179-185 |
| San Felice Acquasparta 1 | 212-212 | 212-212 | 130-144 | 170-170 | 144-144 | 150-150 | 166-193 | 154-205 | 205-205 | 198-206 | 177-179 |
| San Felice Acquasparta 2 | 208-212 | 214-214 | 126-144 | 157-157 | 144-144 | 150-150 | 166-193 | 143-154 | 205-232 | 198-206 | 177-179 |
| San Felice Acquasparta 3 | 212-222 | 214-224 | 124-144 | 184-184 | 144-144 | 150-150 | 166-193 | 182-205 | 205-205 | 198-206 | 177-179 |
| San Felice Acquasparta 4 | 212-212 | 214-259 | 130-144 | 159-184 | 140-140 | 150-150 | 166-193 | 182-182 | 220-220 | 198-206 | 177-179 |
| San Felice Acquasparta 5 | 212-212 | 214-259 | 130-144 | 159-184 | 140-140 | 150-150 | 166-193 | 182-182 | 220-220 | 198-206 | 177-179 |
| San Francesco | 208-208 | 210-210 | 126-130 | 170-184 | 140-140 | 150-150 | 156-182 | 182-182 | 213-213 | 182-206 | 179-181 |
| San Lazzero | 208-212 | 214-224 | 124-144 | 159-184 | 144-144 | 150-150 | 177-177 | 182-205 | 205-205 | 172-194 | 179-181 |
| Sanginara | 214-222 | 218-218 | 124-126 | 150-157 | 140-140 | 143-143 | 164-177 | 154-161 | 205-205 | 182-206 | 181-185 |
| **Santa Caterina** | **208-208** | **210-212** | **124-144** | **136-197** | **150-150** | **143-143** | **156-164** | **150-182** | **220-220** | **162-194** | **185-185** |
| Santa Maria | 214-218 | 214-218 | 124-144 | 157-159 | 140-150 | 143-143 | 164-177 | 154-205 | 205-205 | 172-172 | 173-179 |
| Sant'Agatese | 208-222 | 214-214 | 124-126 | 150-184 | 144-144 | 150-150 | 177-177 | 182-182 | 213-213 | 172-182 | 177-179 |
| Sant'Agostino | 212-212 | 210-214 | 124-144 | 170-170 | 144-144 | 143-143 | 166-166 | 154-154 | 108-108 | 172-198 | 177-181 |
| Santomauro | 208-222 | 214-214 | 124-144 | 157-184 | 144-150 | 143-143 | 166-182 | 143-205 | 205-232 | 182-198 | 177-179 |
| Sargano | 214-222 | 218-224 | 124-144 | 159-184 | 144-144 | 143-143 | 177-182 | 182-210 | 185-185 | 182-198 | 179-181 |
| Sargano | 218-222 | 214-214 | 130-144 | 150-157 | 150-150 | 135-135 | 166-177 | 154-205 | 200-205 | 172-182 | 177-177 |
| Scarpetta | 208-218 | 212-221 | 124-124 | 150-159 | 144-144 | 150-150 | 177-182 | 143-143 | 200-200 | 172-198 | 179-181 |
| Selvatico | 214-222 | 214-214 | 126-144 | 150-157 | 150-150 | 135-135 | 177-193 | 161-210 | 213-232 | 172-182 | 177-181 |
| Semidana | 214-214 | 214-218 | 130-144 | 136-184 | 144-150 | 135-135 | 164-193 | 150-161 | 200-200 | 162-172 | 181-185 |
| Sessana | 214-214 | 214-224 | 126-130 | 170-184 | 144-144 | 143-143 | 166-177 | 182-205 | 213-213 | 182-206 | 181-185 |
| Simona | 212-222 | 210-210 | 124-126 | 159-159 | 144-144 | 166-166 | 166-177 | 154-154 | 108-170 | 182-182 | 175-181 |
| Sinopolese | 212-214 | 212-212 | 124-130 | 170-184 | 144-144 | 143-143 | 177-193 | 154-210 | 164-205 | 162-198 | 177-179 |
| Sirole | 214-222 | 218-224 | 130-144 | 150-157 | 140-140 | 135-135 | 177-182 | 182-210 | 185-220 | 176-206 | 181-185 |
| Sivigliana da olio | 214-222 | 214-214 | 130-130 | 159-159 | 150-150 | 135-135 | 166-177 | 210-210 | 213-232 | 162-176 | 181-181 |
| Spagnola di Missano | 208-208 | 212-224 | 124-144 | 157-159 | 144-144 | 143-143 | 166-177 | 143-210 | 185-185 | 176-198 | 181-185 |
| Sperone di Gallo | 212-212 | 214-214 | 130-130 | 170-170 | 140-140 | 143-143 | 166-193 | 182-182 | 213-213 | 162-172 | 169-179 |
| Spezzanese | 212-222 | 214-214 | 124-130 | 136-170 | 144-144 | 143-143 | 166-193 | 143-154 | 205-205 | 162-198 | 179-185 |
| Spinoso | 208-222 | 214-214 | 126-144 | 159-184 | 144-144 | 150-150 | 166-182 | 143-210 | 205-213 | 194-198 | 179-181 |
| Taggiasca | 208-212 | 214-224 | 124-144 | 159-170 | 144-144 | 143-143 | 177-182 | 182-205 | 205-205 | 172-194 | 179-181 |
| Tenacella | 218-218 | 214-218 | 126-144 | 157-159 | 140-150 | 143-143 | 164-164 | 161-161 | 213-213 | 172-206 | 181-181 |
| Tendellone | 214-222 | 218-221 | 126-144 | 159-159 | 140-140 | 150-150 | 166-182 | 143-182 | 205-205 | 176-198 | 179-181 |
| Termite di Bitetto | 208-218 | 214-214 | 124-144 | 150-159 | 144-144 | 143-143 | 177-182 | 143-205 | 108-170 | 172-194 | 179-179 |
| Terza grande | 214-222 | 214-221 | 126-144 | 157-159 | 144-150 | 143-143 | 164-177 | 150-161 | 213-213 | 182-198 | 179-181 |
| Terza piccolo | 214-222 | 212-218 | 126-144 | 157-159 | 144-150 | 143-143 | 164-177 | 150-161 | 213-213 | 176-182 | 179-181 |
| **Toccolana** | **212-222** | **214-214** | **124-130** | **150-150** | **144-144** | **150-182** | **177-193** | **154-205** | **205-205** | **162-188** | **173-183** |
| Tombarello | 212-212 | 212-212 | 130-144 | 150-159 | 150-150 | 143-143 | 177-193 | 143-182 | 205-205 | 172-206 | 177-177 |
| Tonda di Alife | 214-214 | 214-214 | 124-126 | 150-157 | 150-150 | 166-166 | 164-193 | 150-154 | 205-205 | 182-206 | 181-185 |
| Tonda di Cagliari | 212-212 | 214-221 | 126-144 | 170-203 | 150-150 | 135-135 | 166-193 | 143-143 | 213-220 | 162-172 | 185-185 |
| Tonda di Filadelfia | 212-222 | 212-218 | 126-144 | 150-157 | 144-144 | 143-143 | 166-193 | 143-205 | 205-232 | 162-198 | 177-185 |
| Tonda di Filogaso | 212-212 | 212-250 | 124-124 | 157-170 | 150-150 | 143-143 | 166-166 | 143-182 | 205-205 | 198-206 | 177-179 |
| Tonda di Strongoli | 212-222 | 212-218 | 124-126 | 136-150 | 144-144 | 143-143 | 166-193 | 143-182 | 205-220 | 162-198 | 177-185 |
| Tonda Dolce | 208-208 | 214-214 | 126-144 | 150-159 | 144-144 | 143-143 | 166-193 | 143-154 | 170-170 | 162-198 | 177-185 |
| Tonda Dolce di Partanna | 212-212 | 214-214 | 144-144 | 157-184 | 140-140 | 150-150 | 166-193 | 182-182 | 205-205 | 182-182 | 177-181 |
| Tonda iblea | 208-212 | 210-214 | 124-144 | 136-159 | 144-144 | 150-150 | 166-193 | 154-154 | 108-205 | 162-198 | 169-177 |
| Tondina | 208-222 | 214-214 | 126-144 | 159-159 | 150-150 | 143-143 | 166-182 | 143-182 | 205-232 | 198-206 | 177-179 |
| Tortiglione | 208-212 | 214-224 | 124-126 | 159-170 | 144-144 | 166-166 | 177-177 | 154-210 | 220-243 | 198-206 | 179-181 |
| Toscanina | 218-218 | 210-214 | 124-144 | 150-159 | 144-144 | 143-143 | 177-177 | 161-182 | 164-164 | 172-198 | 177-181 |
| Tunnulidda | 208-212 | 214-214 | 126-144 | 159-184 | 144-144 | 150-150 | 166-193 | 182-182 | 205-205 | 172-182 | 169-181 |
| Turdunazza antimosca 1 | 212-212 | 210-214 | 126-144 | 159-184 | 140-140 | 150-150 | 166-193 | 143-143 | 213-232 | 172-172 | 173-179 |
| Turdunazza antimosca 2 | 212-212 | 210-214 | 126-144 | 159-184 | 140-140 | 150-150 | 166-193 | 143-143 | 213-232 | 172-198 | 179-185 |
| Uccellara | 212-212 | 212-212 | 124-144 | 159-170 | 144-144 | 143-143 | 166-177 | 143-143 | 200-200 | 198-206 | 179-181 |
| Ugea di Savoia | 212-212 | 212-212 | 126-130 | 159-184 | 140-140 | 143-143 | 166-166 | 143-161 | 205-205 | 162-172 | 177-181 |
| Ugliastride | 208-218 | 214-221 | 124-144 | 170-184 | 140-150 | 143-143 | 166-193 | 143-182 | 200-200 | 172-176 | 185-185 |
| Vaddara | 208-212 | 210-214 | 124-124 | 157-159 | 144-144 | 150-150 | 177-193 | 154-182 | 164-205 | 198-198 | 179-179 |
| Vallanella 1 | 208-212 | 214-224 | 130-144 | 170-170 | 144-144 | 150-150 | 166-193 | 143-210 | 213-232 | 162-206 | 173-175 |
| Vallanella 2 | 208-212 | 210-224 | 124-144 | 157-157 | 144-144 | 150-150 | 166-193 | 154-154 | 205-205 | 162-206 | 173-175 |
| Verdello | 212-222 | 210-214 | 124-126 | 159-170 | 144-144 | 150-150 | 166-166 | 143-143 | 170-220 | 182-206 | 181-181 |
| Vigna della Corte | 212-214 | 214-218 | 124-144 | 157-159 | 140-140 | 143-150 | 164-177 | 150-154 | 213-220 | 182-206 | 179-181 |
| Vocio | 214-214 | 214-214 | 130-144 | 150-157 | 140-140 | 143-143 | 166-177 | 161-205 | 205-213 | 198-206 | 177-181 |
| Vraja | 208-218 | 214-214 | 126-144 | 170-184 | 144-144 | 143-143 | 166-193 | 143-182 | 200-200 | 162-198 | 179-185 |
| Zaituna | 212-212 | 214-214 | 124-144 | 150-170 | 140-140 | 150-150 | 166-193 | 182-182 | 108-108 | 182-206 | 177-177 |
| Zimbimbo | 208-218 | 214-224 | 124-144 | 157-170 | 144-144 | 135-135 | 177-182 | 182-182 | 108-108 | 198-198 | 177-179 |
| Zinzifarica | 212-222 | 214-214 | 126-144 | 150-159 | 144-144 | 143-143 | 177-177 | 143-182 | 205-205 | 162-198 | 177-179 |
